# Engineering High-Performance Carbazole-Based Co-Sensitizers: Synthesis, Photophysical Characterization, and Synergistic Enhancement in Dye-Sensitized Solar Cells

**DOI:** 10.1007/s10895-025-04194-1

**Published:** 2025-03-06

**Authors:** Mariam Eltoukhi, Safa A. Badawy, Ahmed A. Fadda, Ehab Abdel-Latif, Mohamed R. Elmorsy

**Affiliations:** 1 Department of Chemistry, Faculty of Science, Mansoura University, El-Gomhoria Street, Mansoura, 35516 Egypt; 2 Department of Chemistry, Faculty of Science, New Mansoura University, New Mansoura, 35712 Egypt

**Keywords:** Dye-sensitized solar cells (DSSCs), Carbazole-based sensitizers, Co-sensitization, Photophysical properties, Power conversion efficiency

## Abstract

The current research implies the synthesis of two novel organic co-sensitizers based on carbazole, which are referred to as **MA-1** and **MA-2**. The performances of these sensitizers in dye-sensitized solar cells (DSSCs) were also studied. The molecular structures were designed using donor-π-acceptor (D-π-A) configurations, where 9-heptyl carbazole and 9-ethyl carbazole served as donors and malononitrile (**MA-1**) and cyanoacetic acid (**MA-2**) acted as acceptor/anchoring groups. The photophysical features of the sensitizers were evaluated via UV-Vis spectroscopy, revealing broad absorption in the visible range with *λ*_*max*_ at 468 nm (**MA-1**) and 478 nm (**MA-2**) and corresponding molar extinction coefficients of 3.76 × 10⁴ M⁻¹cm⁻¹ and 5.34 × 10⁴ M⁻¹cm⁻¹, respectively. Electrochemical analysis showed favorable ground state oxidation potentials (GSOP) and excited state oxidation potentials (ESOP) for both dyes, with optical band gaps of 2.44 eV (**MA-1**) and 2.28 eV (**MA-2**). These values indicate efficient electron injection into the TiO_2_ conduction band and thermodynamic feasibility for dye regeneration. Co-sensitization using the benchmark **N3** dye significantly enhanced the photovoltaic performance. The (**MA-2** + **N3**) achieved the highest power conversion efficiency (*PCE*) of 9.82%, with a short-circuit current density (*J*_*SC*_) of 23.91 mA/cm², an open-circuit voltage (*V*_*OC*_) of 685 mV, and a fill factor (*FF*) of 0.60. This superior performance can be attributed to the synergistic interactions between the dyes, broader spectral coverage, and improved charge separation dynamics. By utilizing synergistic co-sensitization strategies, these findings shed light on the capacity of carbazole-based co-sensitizers, particularly **MA-2**, as potential candidates for high-efficiency DSSCs.

**Supplementary Information:**

The online version contains supplementary material available at 10.1007/s10895-025-04194-1.

## Introduction

**Fig. 1:**
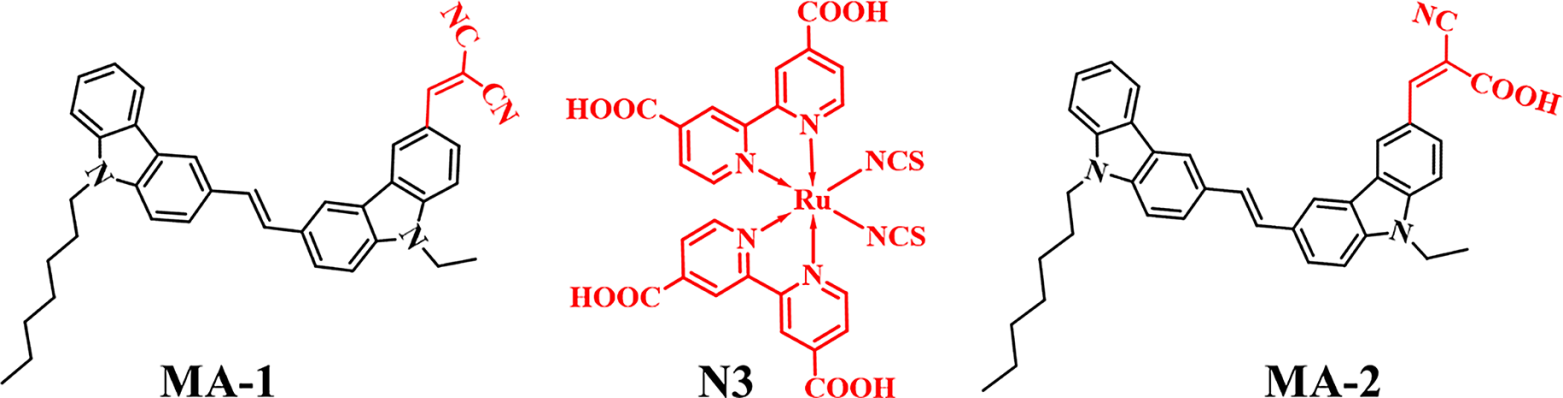
Molecular structures of co-sensitizers **MA-1-2** and **N3**

Solar power, which does not release harmful byproducts into the atmosphere, is one of the most promising sources of clean energy because its supply is both abundant and environmentally friendly. Dye-sensitized solar cells (DSSCs) are gaining popularity due to their cost-effectiveness and ease of fabrication compared to silicon-based photovoltaic modules, which, despite their widespread application, remain expensive [1, 2]. One of the main ideas behind dye-sensitive solar cells is to begin with a thin layer of nanocrystalline TiO_2_. Following this, a photosensitizer, also known as a dye, should have an extremely high molar extinction coefficient to carry out chemical attachment to the surface. This served as the working electrode of the solar cell. A liquid electrolyte containing iodide and triiodide separates the working electrode from the platinum counter electrode [3]. Recent practical and theoretical investigations have focused on the advancement of dyes, which helps boost the effectiveness of (DSSCs) [4]. Dyes are often categorized into two types: metal-based and metal-free. Metal-based dyes, including ruthenium-based-dyes Ru (II), such as **N3** and **N-719**, play an essential role in dye-sensitized solar cell applications, achieving photovoltaic efficiencies of over 10%, owing to their extensive absorption coefficients [5]. The limited absorption of (MLCT) band, in addition to the expensive expenditure on Ru (II) complexes, are common problems despite their increased efficiency [6]. The remarkable light-harvesting and durability properties associated with such photosensitizers can be ascribed to a process called (MLCT), which facilitates the rapid movement of photoelectric charges toward TiO_2_, exceeding electron recombination using the oxidized dye component, rather than passing through the circuit [7, 8]. Moreover, metal-free organic sensitizers are preferred over Ru (II)-based sensitizers because of their numerous advantages, including versatile structure, economical manufacture, and enhanced molar extinction coefficients, which facilitate intramolecular charge transfer (ICT) between an electron-rich donor and an anchoring section via the use of a π-spacer when light is absorbed [9, 10]. Co-sensitization is considered a promising technique for boosting the efficiency of DSSCs. The approach described above involves incorporating organic sensitizers (providing visible light) coupled with Ru (II) complexes (producing near-infrared light) to achieve extensive spectrum sensitivity within the region of visible light and to enhance light absorption [11]. The following are expected to satisfy the requirements for improved DSSC performance. The photovoltaic performance is enhanced when DSSCs are co-sensitized rather than when only one sensitizer is used [12, 13]. The reasons for this include the lack of dye aggregation, low charge recombination, and high molar extinction coefficients, all of which allow the cell to absorb as many incoming photons as possible. Co-sensitizers, to fill in the surface area that the larger sensitizers leave behind, should be on the smaller side [14]. A popular adsorption sensitizer on nanocrystalline TiO_2_ surfaces for collecting and transporting photoelectrons is the ruthenium complex, also known as **N3** dye. Unidentate, bridging bidentate, and chelating are three types of coordination modes that allow the carboxylate groups of **N3** dye to chemically attach themselves to the surface of titanium. **The N3** dye has been widely proven to possess a higher photovoltaic performance. Furthermore, the mesoporous structure of the nanocrystalline TiO_2_ anode played an essential role in promoting dye diffusion and adsorption, facilitating incident light flow and brightness, and increasing the efficiency of electron transmission. **N3** has a conversion efficiency of 10.3% [15]. Therefore, it has been proven that co-sensitization with additional dyes for panchromatic engineering is a significant and successful technique to increase the visible light capture capabilities of DSSCs [16]. A π-spacer often links a donor (D) and an acceptor (A) in the classical pattern associated with organic dyes. During photoexcitation, intramolecular charge separation and transfer are induced by the (D-π-A) A configuration. Donor groups often utilized in D–π–A configurations include carbazole, phenothiazine, indoline, coumarin, and triphenylamine (TPA) [17–20]. Carbazole and its derivatives are recognized for their superior photovoltaic performance, attributed to their effective hole transport capacity, reduced bandgap, and excellent absorption characteristics [21]. Hence, building organic sensitizers with less aggregation propensity is of utmost importance. An effective method was developed by adding alkyl chains to organic patterns. Consequently, this not only reduces the development of aggregates but also increases the molar extinction coefficient of the organic sensitizer [22]. Extending additional donor segments beyond the main donor produces structures recognized as donor-donor-π bridge-acceptors (D-D-π-A), which have a lesser inclination towards aggregation. This is another technique that consider [23]. Likewise, the predominant acceptor employed in the fabrication of organic dyes for (DSSCs) is the cyanoacrylic acid (CCA) moiety. Cyanoacrylic acid is the acceptor that has been the subject of extensive research and continues to be the most effective, as it establishes a robust ester connection with the TiO_2_ surface, facilitating the injection of electrons via the dye towards the conduction band (CB) of TiO_2_. Additionally, it increases the amount of dye loading within the surface of the semiconductor, causing an increase in (*PCE*) of the DSSC compared to utilizing other acceptors [24]. According to the literature, malononitrile is a lower-income acceptor than cyanoacetic acid [25]. In the present study, we constructed carbazole-based organic co-sensitizers (**MA-1-2**) using heptyl carbazole and ethyl carbazole as powerful electron donors and malononitrile and cyanoacrylic acid as electron-withdrawing or anchor moieties. Figure 1 shows a representation of their molecular structures. Heptyl carbazole and ethyl carbazole, when added to the π-bridged system, minimized the bandgap and boosted the electrochemical, optical, and photovoltaic features. Throughout the visible spectrum, which extends from 400 to 600 nm, the co-sensitizers (**MA-1-2**) highlighted an obvious absorption band. When compared with **MA-1**, the co-sensitizer (**MA-2**) reveals the largest ε (5.34 × 10^4^ M⁻^1^ cm⁻^1^), while it additionally has the shortest energy gap (2.28 eV). Based on the research findings, **MA-2** + **N3** sensitizers produced more effective *PCEs* (9.82%) than **N3** (6.25%), **MA-1** (6.95%), **MA-2** (7.84%), and **MA-1** + **N3** (8.85%); therefore, they are excellent choices for DSSC applications. Furthermore, owing to the more powerful electron injection, in addition to a more significant efficiency, (**MA-2** + **N3**) provides the greatest (*J*_*SC*_ = 23.91 mA/cm²).

## Experimental

### Synthesis of 9-heptyl-9*H*-carbazole (2)

After dissolving (0.16 g, 1 mmol) of carbazole (**1**) and (0.56 g, 10 mmol) of Potassium hydroxide in 10 mL of dimethylsulfoxide, the mixture was stirred for 0.5 h at room temperature under argon atmosphere. Subsequently, (0. 314 g, 2 mmol) of 1-bromoheptane was introduced into the aforementioned reaction mixture while stirring continuously for 8 h with heating at 60 °C. Using TLC, the reaction was detected by TLC. After completion of the reaction, 100 mL of cold water was added, and diluted hydrochloric acid (HCl) was used to neutralize it. Ethyl acetate was the solvent of choice for the extraction. The compound 9-heptyl-9*H*-carbazole (**2**) was isolated from the organic phase by washing with water and brine, drying with Na_2_SO_4_, and then filtering. Yield: 85%, colorless oil; mp: 43–45 °C [26].

### Synthesis of 9-heptyl-9*H*-carbazole-3-carbaldehyde (3)

A solution containing 1.68 mL (18 mmol) of freshly distilled POCl_3_ was added dropwise to 1.63 mL (21 mmol) of dry DMF in a 50 mL two-neck RB flask. The resulting mixture was stirred at 0 °C in argon atmosphere to fully precipitate the colored Vilsmeier salt. Subsequently, a solution of (**2**) (0.27 g, 1 mmol) dissolved in 10 mL of dichloroethane solvent was incrementally added to the reaction mixture dropwise while stirring and maintaining a temperature of 60 °C for 5 h. The reaction mixture was then stirred for 48 h at ambient temperature. Upon completion of the reaction, the mixture was poured into 100 mL of ice-cold water, and the pH was adjusted to alkaline by adding a saturated sodium acetate solution, followed by extraction with dichloromethane. The pale-yellow liquid, once viscous, solidified upon standing at room temperature to synthesize the aldehyde (**3**). Yield: 70%, pale yellow solid; mp 63–65 °C; lit. mp: 60–62 °C [27].

### Synthesis of 9-ethyl-9*H*-carbazole-3,6-dicarbaldehyde (6)

Drops of freshly distilled POCl_3_ (15 mL, 160 mmol) were added to a stirred solution of dry DMF (10 mL, 129 mmol) in a 50 mL two-neck RB flask at 0 °C under an argon atmosphere until the colored Vilsmeier salt was completely formed. Gradually, while stirring and maintaining a temperature of 100 °C for 48 h, a solution of 0.50 g (2.60 mmol), of 9-ethyl-9*H*-carbazole was added to the reaction mixture in 10 mL of DMF solvent. It was subsequently poured into a mixture of ice and water, and the pH was elevated to an alkaline level using a saturated sodium acetate solution. An ethyl acetate and petroleum ether mixture was used as the eluent in column chromatography using silica gel, which was utilized to purify the end product. The concentration ratio between the two liquids was 1 2. Yield: 70%, white solid, mp: 173–175 °C; lit. mp: 168–170 °C [28].

### Synthesis of 9-ethyl-6-(2-(9-heptyl-9*H*-carbazol-3-yl) Vinyl)-9*H*-carbazole-3-carbaldehyde (7)

The following ingredients were added: 0.25 g of ethyl-9*H*-carbazole-3,6-dicarbaldehyde 6 (6 mmol), 15 mg of 18-crown-6 (0.05 mmol), and 0.276 g of K_2_CO_3_ (2 mmol). Stirring was continued for 30 min. Add 0.620 g and 1 mmol of adduct **5** in 15 mL of dry DMF dropwise to the mixture. It was then allowed to sit for 3 h at 70 °C with nitrogen. The reaction mixture was heated to 70 °C and stirred for 24 h to allow the completion of the reaction, which was observed by TLC. The mixture was then poured into 100 mL of ice-cold water. A mixture of ethyl acetate and petroleum ether at a concentration ratio of 1:9 was used as the eluent in column chromatography using silica gel to isolate the oily substance, yielding aldehyde **7**. Yield: 65%; Oily Pale yellow. FT-IR (KBr): *ν*_*max*_ 3049 (-CH = C), 2953, 2915, 2850 (-C-H) aliphatic, 1687 (C = O), 1623 (C = C) cm^-1^.^1^H NMR (500 MHz, DMSO-*d*_6_): *δ* 0.70 (t, *J* = 7.50 Hz, 3 H, CH_3_), 1.02–1.14 (m, 8 H, CH_2_CH_2_CH_2_CH_2_), 1.28 (t, *J* = 7.50 Hz, 3 H, CH_3_), 1.65 (m, 2 H, CH_2_), 4.25 (t, *J* = 7.00 Hz, 2 H, CH_2_), 4.39 (q, *J* = 7.00 Hz, 2 H, CH_2_), 7.17 (t, *J* = 7.00 Hz, 1H, Ar-H), 7.40–7.43 (m, 3 H, 1Ar-H and 2 H for CH = CH), 7.45–7.49 (m, 2 H, Ar-H), 7.56 (d, *J* = 9.00 Hz, 1H, Ar-H), 7.67–7.69 (m, 2 H, Ar-H), 7.74 (d, *J* = 9.00 Hz, 1H, Ar-H), 7.95 (d, *J* = 8.50 Hz, 1H, Ar-H), 8.14 (d, *J* = 8.00 Hz, 1H, Ar-H), 8.35 (s, 1H, Ar-H), 8.48 (s, 1H, Ar-H), 8.73 (s, 1H, Ar-H), 10.03 (s, 1H, CHO) ppm. ^13^C NMR (125 MHz, DMSO-*d*_6_): *δ* 13.8, 13.9, 22.0, 26.5, 28.5, 28.6, 31.2, 37.6, 42.4, 109.5, 109.6, 109.8, 110.1, 118.2, 118.4, 119.0, 120.4, 122.2, 122.5, 122.6, 123.0, 124.1, 124.5, 125.5, 125.9, 126.3, 126.9, 127.7, 128.4, 128.7, 130.4, 139.7, 139.8, 140.5, 143.6, 192.0 (CHO) ppm. Mass analysis (m/z, %): 512 (M, 19.42), 497 (41.07), 365 (33.64), 284 (41.38), 149 (66.20), 95 (34.54), 77 (33.54), 71 (75.31), 68 (41.16), 55 (100.00), 42 (58.64). Analysis for C_36_H_36_N_2_O (512.70): Calculated: C, 84.34; H, 7.08; N, 5.46%. Found: C, 84.32; H, 7.17; N, 5.56%

### Synthesis of 2-((9-ethyl-6-(2-(9-heptyl-9*H*-carbazol-3-yl) Vinyl)-9*H*-carbazol-3-yl) Methylene) Malononitrile (MA-1)

Refluxed for 10 h, the mixture of 9-ethyl-6-(2-(9-heptyl-9*H*-carbazol-3-yl) vinyl)-9*H*-carbazole-3-carbaldehyde (**7**) (2.01 g, 4 mmol), malononitrile (**8**) (0.26 g, 4 mmol), ammonium acetate (0.31 g, 4 mmol), and 20 mL glacial acetic acid. After the reaction mixture was allowed to cool to ambient temperature, it was poured into cold water. The obtained oily red product was purified by column chromatography using silica gel and petroleum ether-ethyl acetate (3:1) as the eluent to give **MA-1** as an orange-colored solid. Yield: 69%, mp: 288–290 °C. FT-IR (KBr): ν_*max*_ 3016 (-CH = C), 2955, 2922, 2850 (C-H) aliphatic, 2219 (C ≡ N), 1624 (C = C) cm^− 1^.^1^H NMR (400 MHz, DMSO-*d*_6_): *δ* 1.41 (t, *J* = 5.20 Hz, 3 H, CH_3_), 1.74–1.81 (m,11 H, CH_2_CH_2_CH_2_ CH_2_CH_3_), 2.06–2.12 (m, 2 H, CH_2_), 4.52 (t, *J* = 6.00 Hz, 2 H, CH_2_), 4.70–4.72 (m, 2 H, CH_2_), 7.56–7.65 (m, 5 H, Ar-H), 7.67–7.74 (m, 4 H, Ar-H), 7.77–7.76 (m, 1H, Ar-H), 7.86–7.88 (m 2 H, Ar-H), 8.06 ( s, 1H, Ar-H), 8.22–8.24 (m, 2 H, Ar-H), 8.35 (s, 1H, Ar-H) ppm. ^13^C NMR (125 MHz, DMSO-*d*_6_): *δ* 13.3, 14.0, 22.9, 27.5, 27.9, 29.0, 31.6, 42.6, 47.4, 80.4, 104.0, 104.5, 104.6, 109.2, 115.0, 115.6, 120.2, 121.9, 123.9, 124.4, 125.6, 125.9, 126.1, 126.1, 126.5, 126.8, 127.5, 127.6, 128.2 (2 C), 128.4, 128.7, 129.4, 130.1, 139.7, 140.3, 141.1, 141.2, 158.2 ppm. Mass analysis (m/z, %): 560 (M, 20.47), 549 (58.77), 541 (49.15), 431 (58.80), 418 (48.20), 373 (100.00), 356 (60.28), 335 (54.61), 265 (43.17), 233 (44.25), 56 (42.52). Analysis for C_39_H_36_N_4_ (560.75): Calculated: C, 83.54; H, 6.47; N, 9.99%. Found: C, 83.61; H, 6.92; N, 9.96%.

### Synthesis of 2-Cyano-3-(9-ethyl-6-(2-(9-heptyl-9*H*-carbazol-3-yl) Vinyl)-9*H*-carbazol-3-yl) Aacrylic Acid (MA-2)

In a dry 50 mL RB flask, 9-ethyl-6-(2-(9-heptyl-9*H*-carbazol-3-yl) vinyl)-9*H*-carbazole-3-carbaldehyde (**7**) (2.01 g, 4 mmol) and 2-cyanoacetic acid (**9**) (0. 34 g, 4 mmol) was solubilized in glacial acetic acid (15 mL). To the reaction mixture, excess ammonium acetate (0.31 g, 4 mmol) was added and then refluxed for 15 h. The mixture was cooled to room temperature, and the precipitate was collected, filtered, and purified by column chromatography on silica gel using petroleum/ether ethyl acetate (1:1) as the eluent to produce **MA-2** as a dark orange powder. Yield: 73%, mp: Above 300 °C. FT-IR (KBr): ν_*max*_ 2955, 2923, 2849 (C-H) aliphatic, 2220 (C ≡ N), 1667 (C = O), 1626 (C = C) cm^− 1^.^1^H NMR (500 MHz, DMSO-*d*_6_): *δ* 0.80 (br. s, 3 H, CH_3_), 1.18–1.36 (m, 11 H, CH_2_CH_2_CH_2_ CH_2_CH_3_), 1.76 (m, 2 H, CH_2_), 4.37–4.51 (m, 4 H, CH_2_CH_2_), 7.24–7.33 (m, 1H, Ar-H), 7.48–7.58 (m, 5 H, Ar-H), 7.71–7.87 (m, 4 H, Ar-H), 8.22–8.37 (m, 3 H, Ar-H), 8.46 (d, *J* = 14.80 Hz, 2 H, C = CH), 8.91 (br. s, 1H, Ar-H) ppm. ^13^C NMR (125 MHz, DMSO-*d*_6_): *δ* 14.3, 14.35, 2.46, 26.9, 28.9, 29.0, 31.6, 38.0, 42.7, 98.9, 109.9, 110.7, 110.8, 117.8, 118.6, 118.8, 119.3, 120.8, 122.6, 122.9, 123.0, 123.0, 123.2, 124.9, 125.6, 125.8, 126.0, 126.3, 126.6, 127.4, 128.1, 128.6, 129.0, 130.9, 140.0, 140.8, 143.0, 155.6, 164.6 (COOH) ppm. Mass analysis (m/z, %): 579 (M, 16.13), 577 (50.73), 393 (53.15), 311 (51.76), 308 (54.00), 240 (67.81), 148 (100.00), 100 (82.33), 96 (62.30), 91 (65.45), 77 (53.32), 42 (44.19). Analysis for C_39_H_37_N_3_O_2_ (579.74): Calculated: C, 80.80; H, 6.43; N, 7.25%. Found: C, 80.85; H, 6.41; N, 7.33%.

## Results and Dissuasion

### Synthesis

**Fig. 2:**
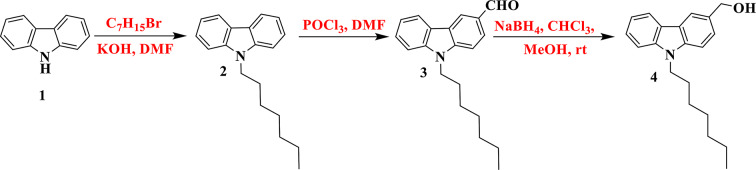
Synthesis of 9-heptyl-9*H*-carbazol-3-yl) methanol (**4**)

Scheme 3 depicts the synthesis of novel new metal-organic dyes (**MA-1-2**). The first step involved synthesizing the intermediate 9-heptyl-9*H*-carbazole (**2**) from 9*H*-carbazole (**1**). Compound **2** was then formylated using the Vilsmeier-Hack reaction procedure, yielding 9-heptyl-9*H*-carbazole-3-carbaldehyde (**3**) [26, 27]. Substance (**4**) 9-heptyl-9*H*-carbazol-3-yl methanol was produced from 9-heptyl-9*H*-carbazole-3-carbaldehyde (**3**) by stirring it for 7 h in a solvent mixture of chloroform and methanol with sodium borohydride. Salt (**5**) 3-((bromotriphenyl-λ^5^-phosphaneyl) methyl)-9-heptyl-9*H*-carbazole was successfully synthesized by the reaction of compound (**4**) with triphenylphosphine hydrobromide and refluxed for 9 h in chloroform as the solvent, as described in (Fig. 2).

**Fig. 3:**
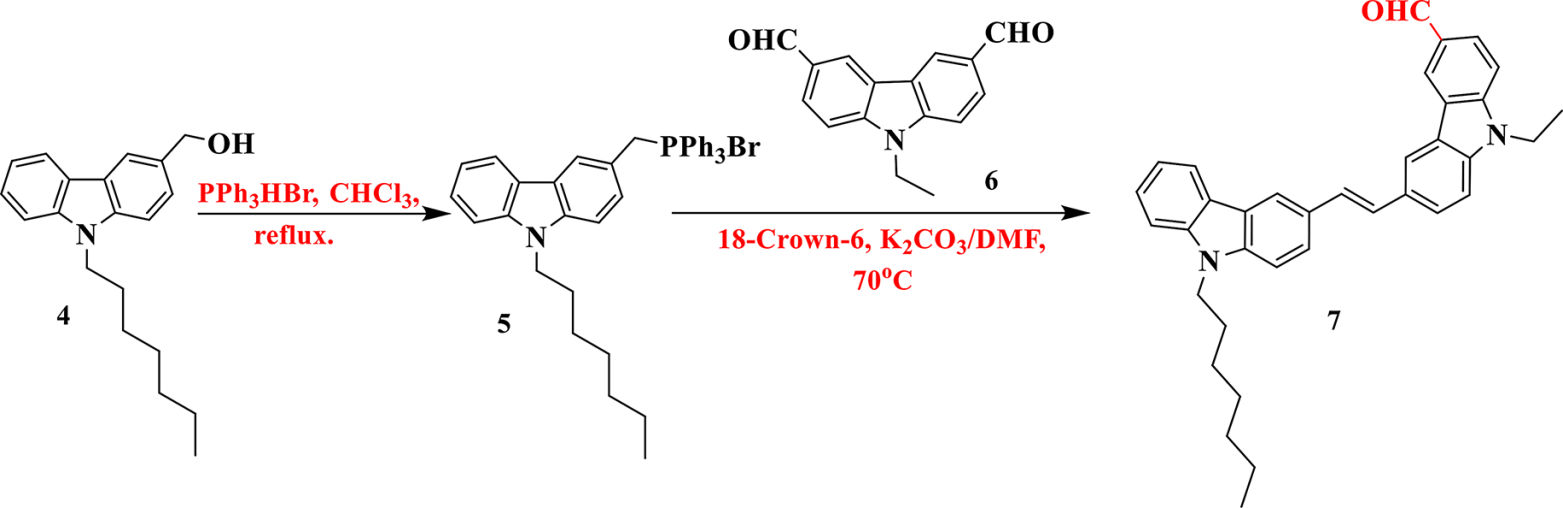
Synthetic routes of carbazole-carbazole aldehyde **7**

Upon solvent removal, the residue was solidified using ether, followed by filtration, to yield compound **5** as a white solid. The synthesis of compound **7** via the Wittig reaction between 3-((bromotriphenyl-λ^5^-phosphaneyl)methyl)-9-heptyl-9*H*-carbazole (**5**) and 9-ethyl-9*H*-carbazole-3,6-dicarbaldehyde (**6**) yielded the requisite oily pale-yellow precursor aldehyde (**7**), as shown in the (Fig. 3).

**Fig. 4:**
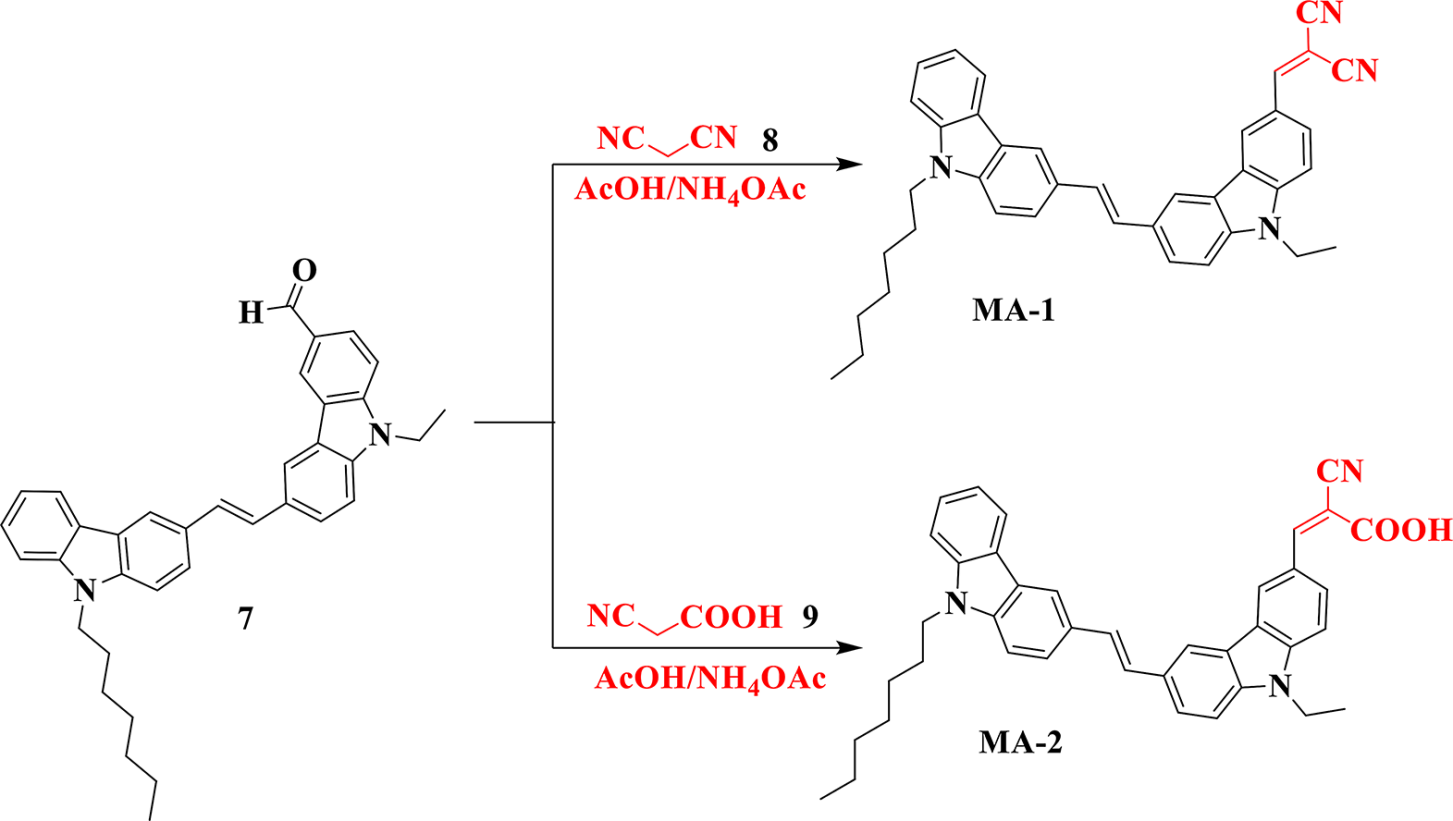
Synthetic routes of co-sensitizers **MA-1-2**

The structure of aldehyde (**7**) was established using specific analytical evaluations. The infrared spectra showed that it had a signature absorption band of the alkyl group at 2953, 2915, and 2850 cm^− 1^ and a pronounced absorption vibration at 1687 cm^− 1^ caused by the C = O group. ^1^H NMR spectra of aldehyde (**7**) exhibited triplet and quartet signals at *δ* 1.28 and *δ* 4.39 ppm, respectively, indicative of the ethyl group (CH_2_CH_3_) of the ethyl carbazole moiety. The heptyl chain of carbazole exhibited a triplet at *δ* 0.70 ppm, a multiplet extending *δ*1.02–1.14 ppm for eight protons, a multiplet for two protons in the range *δ*1.64–1.68 ppm, and a triplet at 4.25 ppm. The aromatic protons manifested as triplet, multiplet, doublet, and singlet signals within the range *δ* 7.17 to 8.739 ppm. The proton of the CHO group exhibits a singlet at *δ* 10.03 ppm. The ^13^ C NMR spectra exhibited distinct signals for nine aliphatic carbons at *δ* 13.85 and 13.94 ppm (2CH_3_) and at 22.07, 26.56, 28.56, 28.67, 31.28, 37.64, and 42.43 ppm (7CH_2_). Moreover, a notable signal at *δ* 192.08 ppm corresponds to the aldehyde group (CHO). Additionally, mass analysis of compound **7** indicated a molecular ion peak at m/z = 512 (19.42%), consistent with the molecular formula C_36_H_36_N_2_O. The target co-sensitizer **MA-1-2** was successfully synthesized in high yield using the Knoevenagel condensation of aldehyde **7** with malononitrile **8** and cyanoacetic acid **9**, utilizing acetic acid as a solvent, and an excess of ammonium acetate, as shown in (Fig. 4).

The co-sensitizer **MA-1-2** and its intermediates were purified using chromatography. Spectroscopic investigations were conducted to verify the structure of the co-sensitizer (**MA-1**). The absorptions recorded in the infrared spectrum at 2955, 2922, 2850, 2219, and 1624 cm⁻¹ correspond to the aliphatic (C-H), cyano (C = O), and vinyl (C = C) groups, respectively. The ^1^H NMR spectrum of **MA-1** manifested a range of signals, including triplet, multiplet signals, extending from *δ* 1.41 to *δ* 4.72 ppm. These signals were associated with the 20 protons that had been detected in the aliphatic area. In addition, the mass spectrum of the substance showed a molecular ion peak at m/z = 560 (20.47%), which was in agreement with the molecular formula (C_39_H_36_N_4_). On the other hand, the chemical structure of the co-sensitizer (**MA-2**) has been verified using elemental analysis and several spectroscopic methods, including IR, ^1^H NMR, ^13^C NMR, and MS. The IR spectra exhibited absorption bands at 2955, 2923, 2849, 2220, 1667, and 1626 cm^− 1^ aligned to the aliphatic (C-H) groups, cyano group (C ≡ N), carbonyl group (C = O), and vinyl groups (C = C), respectively. The carbon of the carboxylic group (COOH) highlighted a signal at *δ* 164.68 ppm in the ^13^C NMR spectrum of compound **MA-2**. In addition, **MA-2’s** molecular formula, C_39_H_37_N_3_O_2_, was confirmed by a molecular ion peak at m/z = 579 (16.13%), as demonstrated by mass analysis.

### Photophysical Properties

**Table 1:** UV–Vis absorption data for co-sensitizers MA-1-2

Sensitizers	λ_max_/ nm	ε/10^4^M⁻^1^ cm⁻^1^	λ_onset_/nm	Experimental E_0 − 0_ (eV)
MA-1	308, 468	1.83, 3.76	507	2.44
MA-2	290, 478	2.26, 5.34	542	2.28

**Fig. 5:**
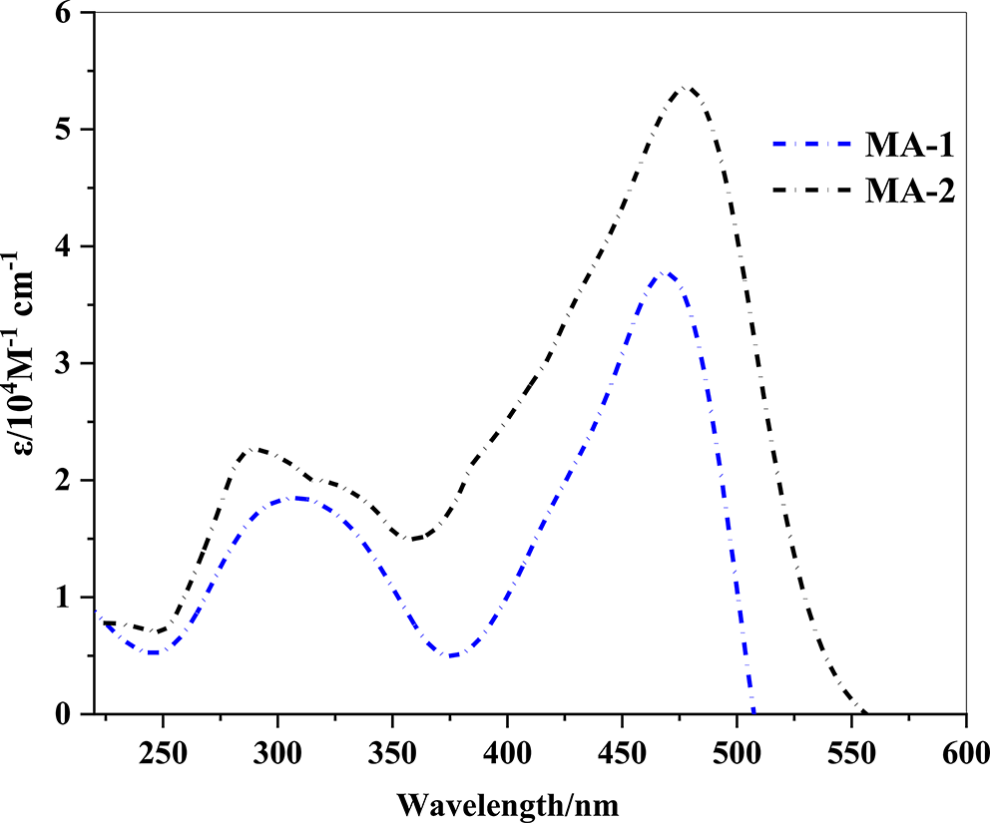
UV–Vis. absorption of **MA-1-2** co-sensitizers

Table 1 shows the UV-Vis absorption spectra of the newly produced co-sensitizer **MA-1-2**. In the presence of a DMF solution, we tested the two synthetic dyes, and the absorption spectra are shown in Fig. 5. All the dyes have two independent absorption bands. The shorter wavelength range (220–350 nm) is represented by one absorption band corresponding to π–π * transitions. The (ICT) phenomenon that occurs between the strong donating portions (9-heptyl carbazole and 9-ethyl carbazole) and the acceptor moieties (malononitrile and cyanoacetic acid) is responsible for the strong absorption that occurs in the visible region from 400 nm to 600 nm. The absorption maxima (*λ*_*max*_) of the new co-sensitizers (**MA-1-2**) have been monitored at 468 and 478 nm, respectively, along with associated molar extinction coefficients (ε) of 3.76 and 5.34 × 10^4^M⁻^1^ cm⁻^1^, implying an increasing order of **MA-2** > **MA-1** as displayed in Table 1. The molar extinction coefficients (ε) of the co-sensitizers (**MA-1-2**) are indeed extremely high in comparison to the Ru dye **N3** (ε = 1.48 × 10^4^M⁻^1^ cm⁻^1^), which indicates more significant light absorption capability [29]. In addition, the onset of optical absorption (UV-visible) could be examined to determine the energy band gap (*E*_*0 − 0*_) [30, 31]. By co-sensitizing N3 with **MA-1** and **MA-2**, a wider range of the solar spectrum was harvested, leading to increased photon absorption and electron injection efficiency. The *E*_*0 − 0*_ measurements for co-sensitizers **MA-1-2** are 2.44 and 2.28 eV, respectively. The diminished delocalization energy of the electron acceptors in the dyes was responsible for the lower *E*_*0 − 0*_ values and greater molar extinction values associated with these co-sensitizers (**MA-1-2**). The absorption spectrum of **MA-2** showed a significant redshift compared to that of **MA-1**. The **MA-2** structure features 9-heptyl carbazole and 9-ethyl carbazole donors as well as cyanoacetic acid, leading to a strong electron-drawing or anchor moiety. Considering the aforementioned, (ICT) between the donor and acceptor portions has the potential to produce a robust charge-separated state in **MA-2**. Furthermore, cyanoacetic acid is composed of two groups (CN and COOH) with extremely conjugated donor parts (9-heptyl carbazole and 9-ethyl carbazole) linked by a π-bridged framework, which minimizes the band gap and increases the light-harvesting characteristics. Co-sensitizer **MA-1** with a malononitrile acceptor and an identical donor component as **MA-2** yields a blue-shifted peak at 468 nm with an approximate ε of 3.76 × 10^4^M⁻^1^ cm⁻^1^; therefore, it confirms malononitrile features a less satisfactory electron withdrawing capacity when compared with **MA-2**.

**Fig. 6:**
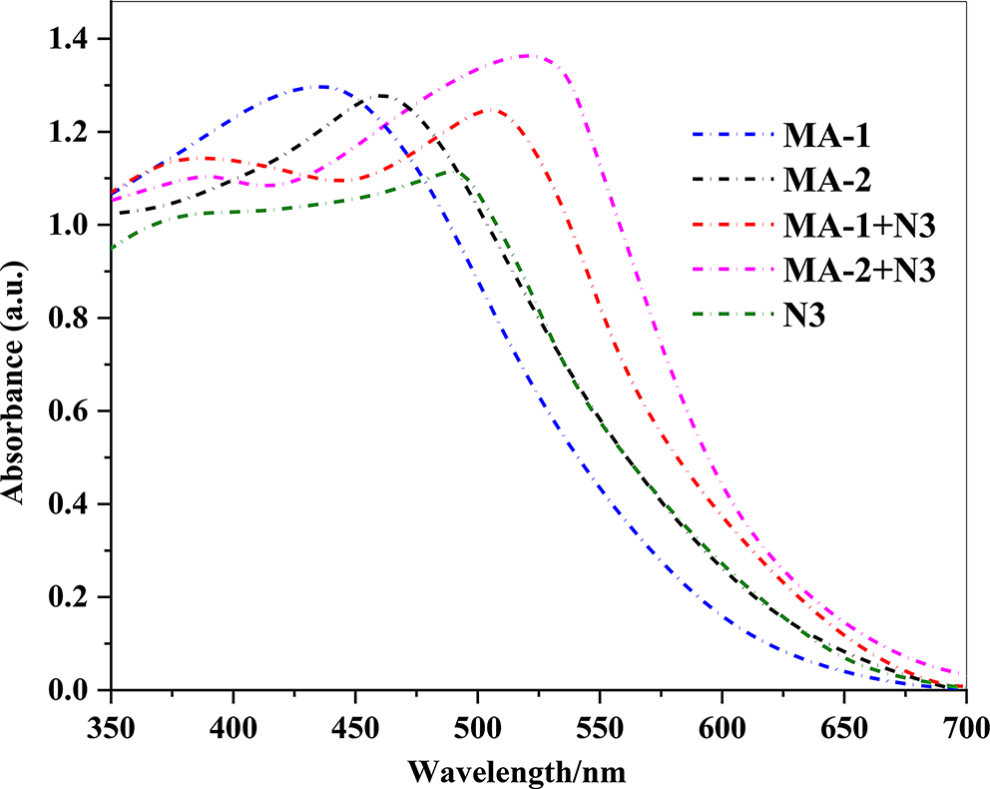
Absorption spectra sensitized and co-sensitized carbazole sensitizers **MA-1-2** with **N3** adsorbed on nonporous TiO_2_

When the absorption spectra of the dyes were compared, **the MA-1-2** co-sensitizers exhibited a wider spectrum in the thin film. This is important for improving the *J*_*SC*_ of the manufactured dyes. In addition, the blue shift observed as an impact of the absorption of the dyes upon sensitization by (**MA-1-2**) onto TiO_2_ sheets is potentially ascribed to dye aggregation of the type *H*-aggregation as well as carboxylic acid deprotonation throughout the surface of the TiO_2_ material (Fig. 6) [32]. Upon analyzing the absorption spectra in Fig. 3, all sensitizers (**MA-1**, **MA-2**, **MA-1** + **N3**, and **MA-2** + **N3**) exhibited higher absorbance and more red shift compared to **N3** alone, indicating higher dye loading on the TiO_2_ surface and *J*-aggregation [33]. This enhanced dye loading is critical for achieving improved light-harvesting efficiency, which directly correlates with (*J*_*SC*_) in DSSCs. The co-sensitization of **N3** with **MA-1-2** dyes further enhanced the spectral response, particularly for **MA-2** + **N3**, which demonstrated the highest absorbance on TiO_2_. The results clearly show that the dye loading of co-sensitization (**MA-1**, **MA-2** + **N3**) outperforms **N3** alone in terms of light-harvesting efficiency, making it a promising candidate for high-performance DSSCs.

### Electrochemical Properties

Cyclic voltammetry measurements were conducted to pinpoint the key electronic parameters for evaluating their suitability with respect to **MA-1** and **MA-2** dyes as sensitizers in DSSC. Table 2 presents the main findings of the voltammograms that were observed, as well as the HOMO-LUMO levels and the estimated band gaps. The experimental (GSOP) energy levels, which represent the HOMO energy levels of the dyes, were − 6.13 eV **MA-1** and − 5.94 eV **MA-2.** The observed values were well below the (CB) of TiO_2_ (-4.20 eV). This implies that these dyes have a favorable energy alignment, which is ideal for electron injection into the TiO_2_ CB. (ESOP) values are − 3.69 eV for **MA-1** and − 3.66 eV for **MA-2**, calculated by applying the Eq. (1), and from CV curve **(S16).**

1 $$\:\varvec{E}\varvec{S}\varvec{O}\varvec{P}=\:-\:\left[\right({E}_{onset}^{oxd}\:+4.7)-\varvec{E}0\--0]\:\varvec{e}\varvec{V}$$

These ESOP values are more negative than the conduction band of TiO_2_. To be certain, ensure the successful implementation of the injection of the electrons generated by the excited dye onto the surface of the TiO_2_ [34, 35]. The experimental (*E*_*0 − 0*_) are 2.44 eV for **MA-1** and 2.28 eV for **MA-2**, providing an adequate extent of the agreement with the theoretical values (2.46 eV and 2.25 eV, respectively). This improved light absorption enhanced photon capture and electron excitation, making **MA-2** a more effective sensitizer. Additionally, the HOMO levels of **MA-1** and **MA-2** were less positive than that of the redox couple (-5.20 eV), facilitating efficient dye regeneration after electron injection [35]. The combination of favorable GSOP, ESOP, and bandgap values demonstrates that both **MA-1** and **MA-2** comply with the thermodynamic conditions necessary for effective electron injection and dye regeneration in DSSCs [35]. Furthermore, the stronger electron-withdrawing character of **MA-2** resulted in slightly lower LUMO energy and enhanced push-pull behavior, which improved the charge separation efficiency within the dye. These results confirm that both **MA-1** and **MA-2** are suitable sensitizer candidates for TiO_2_-based DSSCs, with **MA-2** exhibiting superior light-harvesting capabilities owing to its lower band gap and enhanced electronic properties.

**Table 2:** Optical property values for MA-1-2

Sensitizers	Experimental (eV)			Theoretical (eV)		
	E_0 − 0_	GSOP	ESOP	E_0 − 0_	GSOP	ESOP
MA-1	2.44	-6.13	-3.69	2.46	-6.17	-3.71
MA-2	2.28	-5.94	-3.66	2.25	-5.92	-3.67

### Molecular Modeling for Carbazole Co-Sensitizers MA-1-2

**Fig. 7:**
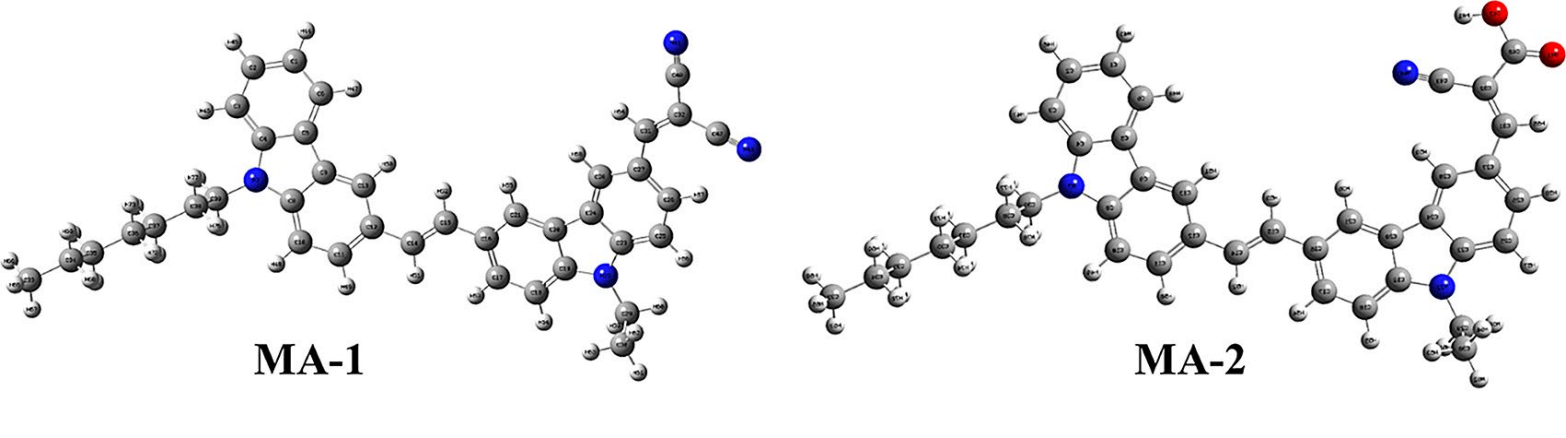
The optimized geometry of co-sensitizers **MA-1-2**

To investigate the electron distribution and equilibrium molecular geometries between the HOMO and LUMO of **MA-1** and **MA-2**, density functional theory (DFT) calculations were conducted using Gaussian 09 software at the B3LYP/6-31G(d, p) level of theory [36]. The optimized molecular structures of the sensitizers (**MA-1-2**) are shown in Fig. 7. These computational studies focused on evaluating the effects of the donor groups heptyl carbazole and ethyl carbazole, as well as the anchoring and acceptor. This analysis provides insight into the electronic and structural features that influence the performance of these co-sensitizers in DSSCs.

**Fig. 8:**
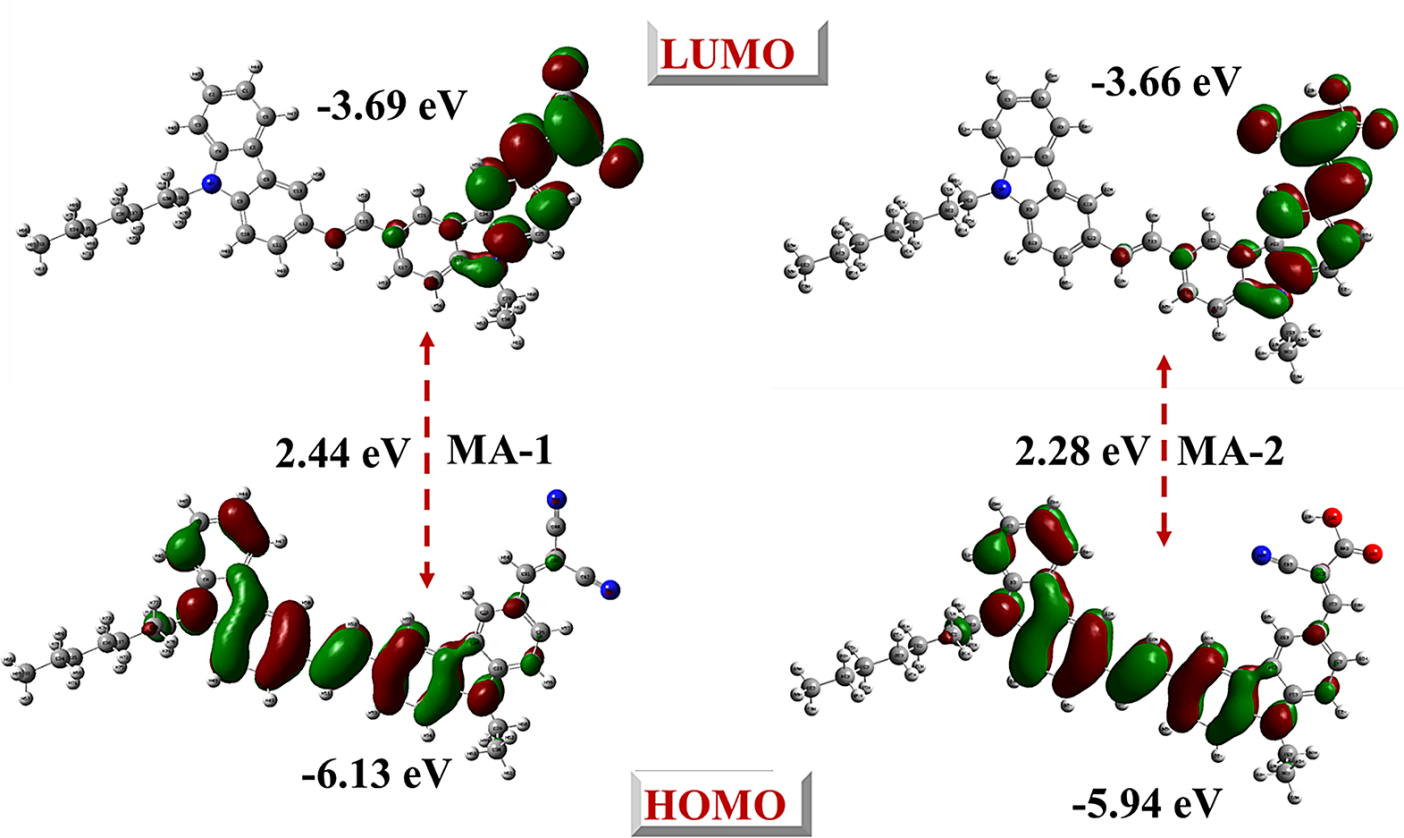
HOMO_S_ and LUMO_S_ for co-sensitizers **MA-1-2**

The performance of the photovoltaic system is extensively linked to the charge separation between the HOMO and LUMO orbitals. As shown in Fig. 8, the HOMO electrons of **MA-**1 and **MA-2** were primarily localized within the donor regions, specifically the 9-heptyl carbazole and 9-ethyl carbazole moieties. In contrast, the LUMO remained concentrated in the acceptor regions, including malononitrile in **MA-1** and cyanoacetic acid in **MA-2**. This distribution facilitates efficient intramolecular movement of electrons through the donor units to the acceptor groups, enhancing electron injection into the TiO_2_ semiconductor surface.

### Molecular Electrostatic Potential (MEP) of Carbazole Co-Sensitizer MA-1-2

**Fig. 9:**
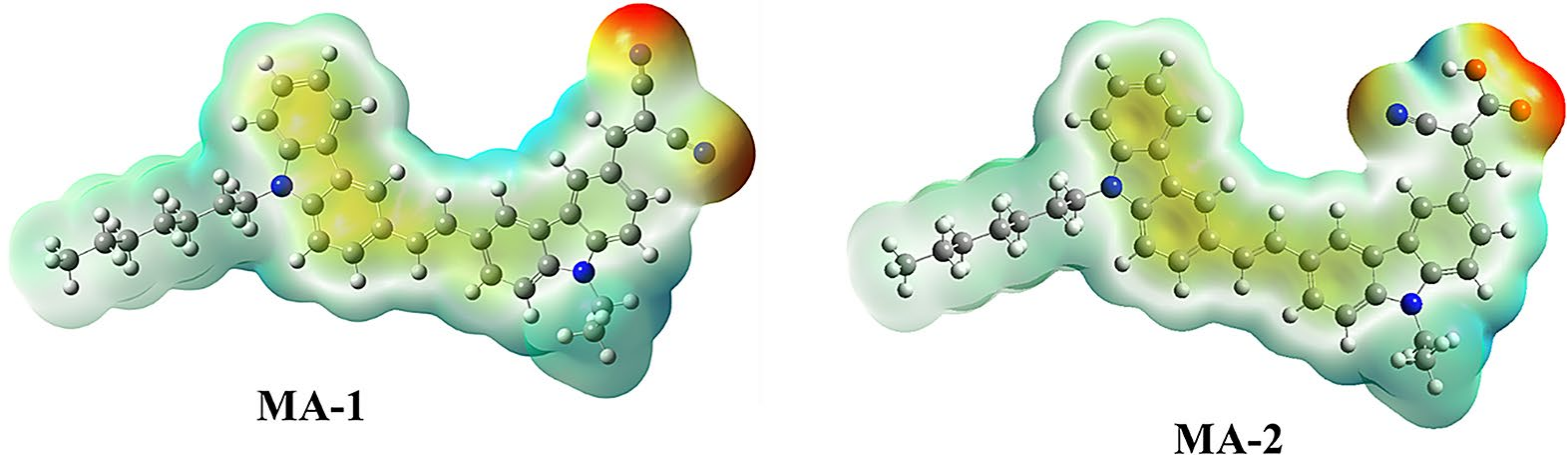
Molecular electrostatic potential for co-sensitizers **MA-1-2**

The capability to properly comprehend the molecular mechanical response of the co-sensitizers **MA-1-2** is a key feature believed to be able to be evaluated utilizing (MEPs) [37]. Researchers can determine the relative impact of donors versus acceptors by studying their dissimilar HOMO-LUMO ratios as well as MEPS on **MA-1-2** (Fig. 9). The red parts of the MEP show the nucleophilic activity associated with areas lacking electrons, whereas the blue parts show the electrophilic activity that corresponds to areas rich in electrons in the co-sensitizer **MA-1-2**. In this sequence, the electrostatic potential increased: red, orange, yellow, green, blue, and ultimately blue. The domain of malononitrile, consisting of **MA-1**’s (CN) and (CN) groups, exhibits the lowest negative low potentials, whereas the co-sensitizer **MA-2** is typically located in cyanoacetic acid (focused on COOH and CN). Nucleophilic attack occurs more probably throughout donor parts, including 9-heptylcarbazole and 9-ethylcarbazole, which are found within the positive zone (blue) of the MEP map. By analyzing **MA-1-2**’s MEP, we can obtain a precise estimation of the number of electrons that can be used in potential interactions with other sets of atoms. The nature of ICT in all **MA-1-2** co-sensitizers adsorbed on TiO_2_ is demonstrated by this new finding.

### TiO2 Electrode Preparation and Device Fabrication

The supplemental information includes specific details regarding the fabrication process and preparation of titanium dioxide (TiO_2_).

### Photovoltaic Device Characterizations

**Fig. 10:**
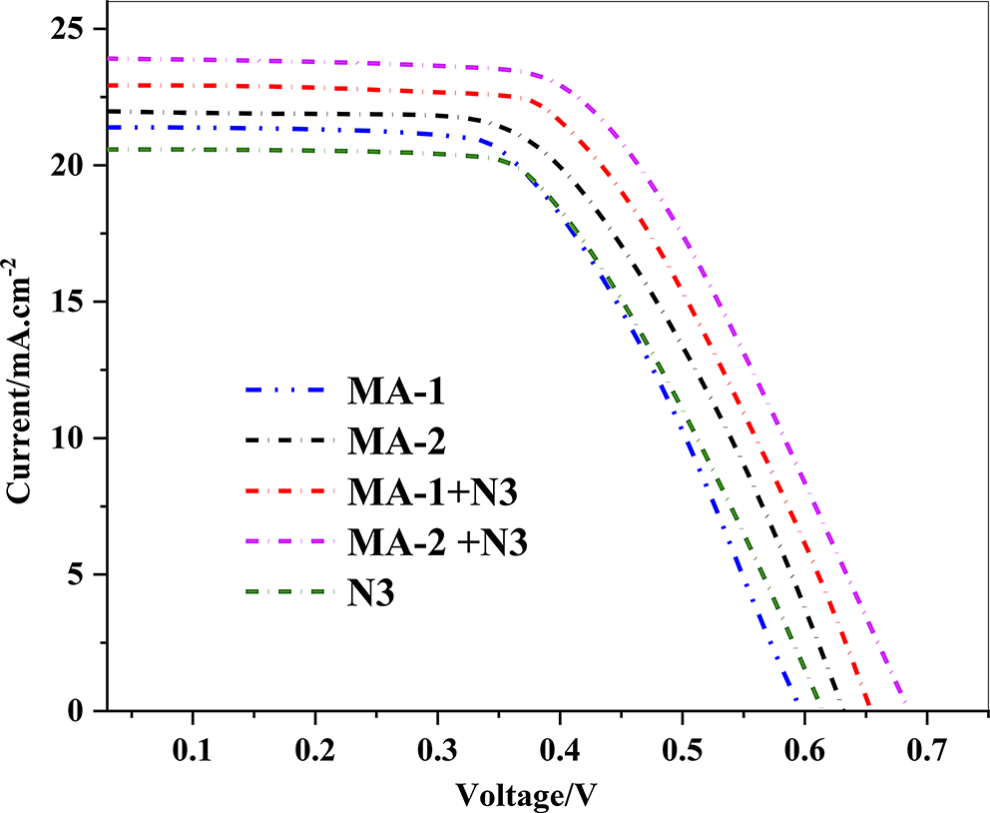
*J-V* curves of sensitized and co-sensitized carbazole sensitizers **MA-1-2** with **N3**

Table 3 provides an overview of the photovoltaic parameters, involving (*J*_*SC*_), (*V*_*OC*_), (*FF*), and (*η*), for dyes **MA-1**, **MA-2**, **N3**, and their co-sensitized systems. The *I-V* curves for these sensitizers, presented in Fig. 10, complement the detailed performance metrics summarized in Table 3. For the sensitizer **MA-1**, which incorporates a malononitrile acceptor group, a (*η*) of 6.95% was recorded, with a *J*_*SC*_ of 21.38 mA/cm², *V*_*OC*_ of 600 mV, and an *FF* of 0.54. This moderate performance can be linked to the relatively weaker electron-withdrawing nature of the malonitrile group, which limits the spectral absorption and reduces the electron injection efficiency into the TiO_2_ conduction band. In comparison, the **MA-2** dye, featuring a cyanoacetic acid acceptor, achieved an enhanced performance of 7.84%, accompanied by a *J*_*SC*_ of 21.97 mA/cm², *V*_*OC*_ of 638 mV, and *FF* of 0.56. The improved performance of **MA-2** is primarily attributed to the stronger electron-withdrawing capacity of the cyanoacetic acid group, which promotes efficient electron injection and mitigates recombination losses [33]. This results in a broader spectral absorption and superior photovoltaic characteristics. The ruthenium-based **N3** dye demonstrated a *PCE* of 6.25%, *J*_*SC*_ of 20.59 mA/cm², *V*_*OC*_ of 608 mV, and *FF* of 0.50. Despite its wider absorption spectrum and effective utilization of photon harvesting inside the red region, **N3’s** lower *V*_*OC*_ and *J*_*SC*_ compared to **MA-1-2** limited its overall efficiency.

**Table 3:** Photovoltaic measurements data of sensitizers MA-1-3 and N3

Sensitizer (0.2mM)	V_OC_^a^(V_OC_^b^)/mV	J_SC_^a^(J_SC_^b^) (mA.cm^− 2^)	FF^a^(FF^b^)/%	PCE^a^ (PCE^b^)/%	Concentration of the dye/10^− 5^mol cm^− 2^
N3	608 (601 ± 6.54)	20.59 (20.43 ± 0.10)	0.50 (0.50 ± 0.01)	6.25 (6.21 ± 0.04)	1.54
MA-1	600 (600 ± 3.68)	21.38 (21.28 ± 0.07)	0.54 (0.50 ± 0.02)	6.95 (6.88 ± 0.06)	1.15
MA-2	638 (624 ± 9.56)	21.97 (21.84 ± 0.09)	0.56 (0.54 ± 0.01)	7.84 (7.77 ± 0.06)	1.34
MA-1 + N3	652 (642 ± 7.13)	23.01 (22.92 ± 0.06)	0.59 (0.56 ± 0.02)	8.85 (8.78 ± 0.05)	1.46
MA-2 + N3	685 (679 ± 3.85)	23.91 (23.84 ± 0.05)	0.60 (0.57 ± 0.01)	9.82 (9.72 ± 0.07)	2.15

^a^The best device parameters (listed in the manuscript)

^b^ The average device parameters (obtained from five devices)

Co-sensitization significantly enhanced device performance. The (**MA-1** + **N3**) system achieved a power conversion efficiency of 8.85%, with a *J*_*SC*_ of 23.01 mA/cm², *V*_*OC*_ of 652 mV, and an *FF* of 0.59. This improvement is prompted by the complementary absorption of **N3** within the red region, as well as **MA-1** in the visible spectrum, coupled with enhanced surface coverage and reduced dye aggregation, leading to more effective charge separation and transport. The highest performance was observed for the (**MA-2** + **N3)** system, which delivered a power conversion efficiency of 9.82%, *J*_*SC*_ of 23.91 mA/cm², *V*_*OC*_ of 685 mV, and *FF* of 0.60. This exceptional performance can be ascribed to the synergistic interaction between **MA-2** and **N3**, where **MA-2**’s cyanoacetic acid group enhances electron injection and **N3** (MLCT) features optimized red-region absorption [33]. This combination provides improved spectral coverage, efficient charge dynamics, and minimized recombination losses, making (**MA-2** + **N3**) the most effective configuration among the tested systems [38].

**Fig. 11:**
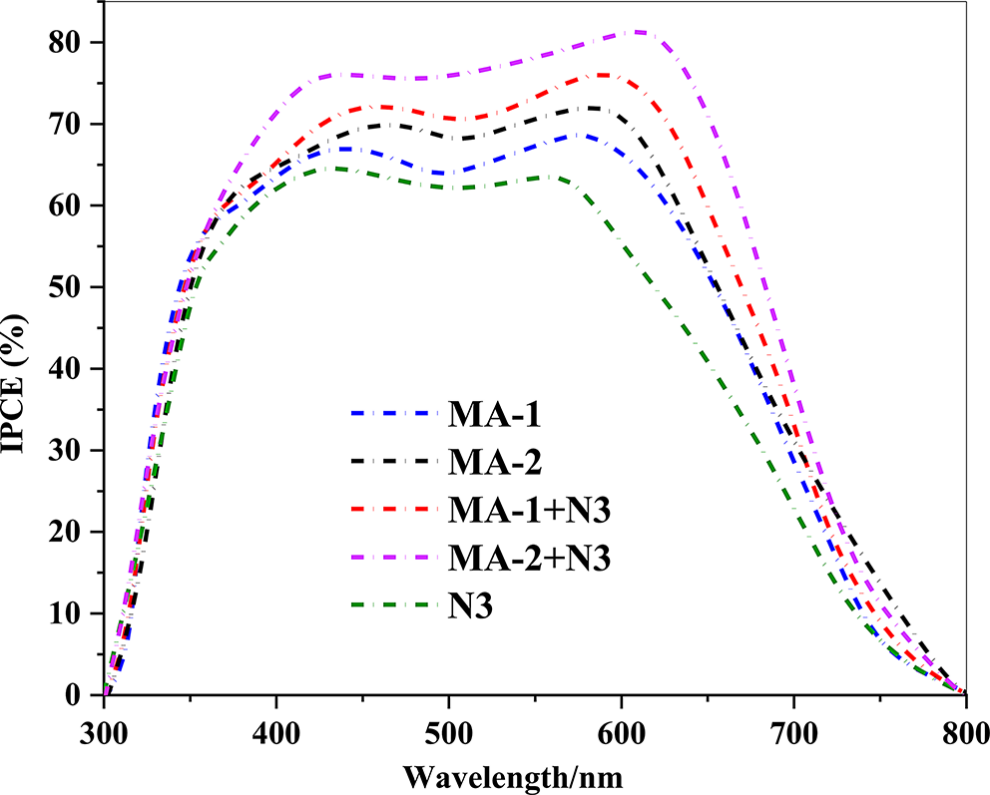
*IPCE* spectra of sensitized and co-sensitized carbazole sensitizers **MA-1-2** with **N3**

The *IPCE* curves provide a detailed comparison of the performance of **MA-1**, **MA-2**, **N3**, and their co-sensitized systems (**MA-1** + **N3** and **MA-2** + **N3**) in DSSCs. **MA-1** achieves an *IPCE* of ~ 68% within the 400–600 nm range, but shows limited efficiency beyond 600 nm owing to its narrower absorption range and weaker electron injection capabilities, as shown in Fig. 11. In contrast, **MA-2** exhibited a slightly higher *IPCE* (70%) and broader spectral coverage, which is attributed to the stronger electron-withdrawing nature of cyanoacetic acid, which enhances electron injection and light absorption [39]. Co-sensitization with **N3** significantly enhanced the performance. (**MA-1** + **N3)** achieved an *IPCE* of 75%, benefiting from complementary absorption in the visible and red regions, and improved surface coverage. (**MA-2** + **N3)** system delivered the highest *IPCE* (80%), attributed to **MA-2’s** efficient electron injection and spectral broadening from cyanoacetic acid combined with **N3’s** strong red-region absorption. This synergistic interaction optimizes light harvesting, reduces recombination losses, and improves charge dynamics. Co-sensitized systems outperform their single-dye counterparts, with (**MA-2** + **N3)** demonstrating the greatest potential for efficient DSSCs through complementary absorption and enhanced electronic properties [40, 41].

## Electrochemical Impedance Spectroscopy (EIS)

**Fig. 12:**
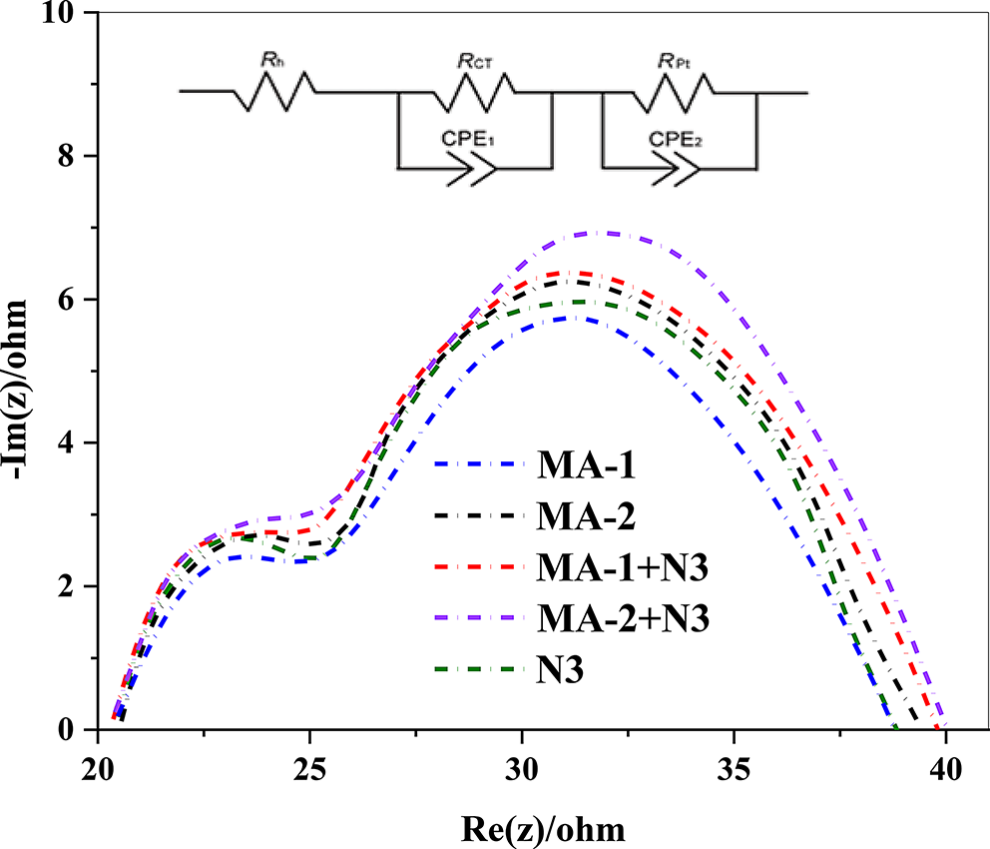
EIS Nyquist plots for sensitized and co-sensitized carbazole sensitizers **MA-1-2** with **N3**

Electrochemical impedance spectroscopy (*EIS*) is a valuable technique for examining interfacial electron charge recombination in DSSCs sensitized with malononitrile and cyanoacetic acid dyes, specifically **MA-1**, **MA-2**, and **N3** [42]. The Nyquist charts indicate that there are two distinct semicircles in various frequency ranges. At higher frequencies, the first semicircle corresponds to the redox resistance to charge transfer at the Pt/electrolyte interface (*R*_*pt*_); along with the lower frequencies, the second semicircle represents the resistance to charge transfer at the TiO_2_/dye/electrolyte interface (*R*_*ct*_), indicating that it is directly connected to (*V*_*OC*_). Based on the results shown in Fig. 12, the order of *R*_*c*t_ throughout the sensitized cells is as follows: **MA-2** + **N3** > **MA-1** + **N3** > **MA-2** > **N3** > **MA-1. **Table 3 lists the recorded *V*_*OC*_ values, which are consistent with these findings. The co-sensitized systems (**MA-1 + N3** and **MA-2 + N3**) exhibited higher *R*_*ct*_ values than the individual dyes, particularly (**MA-2** + **N3)**. This enhancement was attributed to the cyanoacetic acid anchoring group in **MA-2**, which effectively reduced the recombination process. Consequently, for co-sensitization with **MA-2** + **N3**, the improved V_OC_ of the co-sensitized system was due to better coverage of the TiO_2_ surface, reducing the charge recombination.

**Fig. 13:**
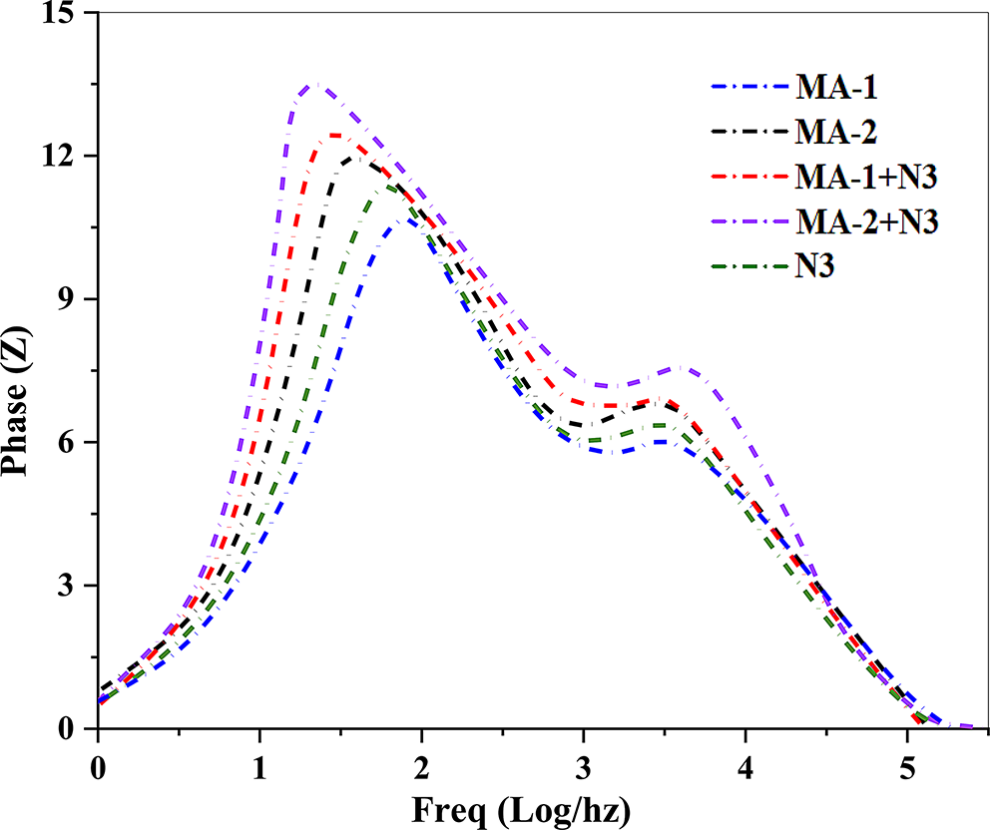
Plots of DSSCs for sensitized and co-sensitized carbazole sensitizers **MA-1-2** with **N3**

The electron lifetime (τ_n_) was calculated from the Bode plots Fig. 13, using τ_n_ = 1/(2πf_max_). The values for DSSCs based on **MA-1**,** MA-2**,** MA-1 + N3**,** MA-2 + N3**, and **N3** were 0.53 ms, 0.64 ms, 0.80 ms, 1.06 ms, and 0.40 ms, respectively. The shortest lifetime for **N3** (τ_n_ = 0.40 ms) indicates severe charge recombination, contributing to its lower *Voc.* Conversely, the longest lifetime for **MA-2 + N3** (τ_n_ = 1.06 ms) reflects reduced recombination, attributed to steric hindrance from its alkyl chains and the strong electron-withdrawing effect of cyanoacetic acid, leading to superior *Voc* and performance. The intermediate τ_n_ of (**MA-1 + N3)** (0.80 ms) highlights the moderate efficiency of malononitrile as an electron acceptor. Overall, co-sensitization significantly enhances τ_n_ reduces recombination, and improves device efficiency, with (**MA-2 + N3)** achieving the best performance.

## Conclusion

In this study, two novel carbazole-based sensitizers, **MA-1** and **MA-2**, with distinct electron acceptor moieties (malononitrile and cyanoacetic acid, respectively) were evaluated for their photophysical and photovoltaic properties. UV-Vis and electrochemical studies demonstrated that both dyes exhibit favorable absorption properties, with **MA-2** displaying a red-shifted absorption peak and a lower optical band gap compared to **MA-1**, indicating superior light-harvesting capabilities. Sensitization by **MA-1-2** achieved a higher efficiency than that by the **N3** metal complex, reaching 6.95% and 7.84%, respectively. Co-sensitization with **N3** dye further enhanced the performance metrics, particularly for the (**MA-2** + **N3)** system, which achieved the highest potential power conversion efficiency (*PCE*) of 9.82%, which was attributed to its synergistic interaction, improved spectral coverage, and enhanced charge dynamics. These findings underline the importance of optimizing dye structures, such as incorporating stronger electron-withdrawing acceptors, such as cyanoacetic acid, to achieve superior efficiency in DSSCs. The co-sensitization strategy demonstrated herein offers a promising route for improving the efficiency of organic and hybrid sensitizers in solar energy applications. The results establish (**MA-2** + **N3**) as a highly effective co-sensitization system, paving the way for future developments in cost-effective, high-performance DSSCs.

## Electronic Supplementary Material

Below is the link to the electronic supplementary material.

Supplementary Material 1

## Data Availability

No datasets were generated or analysed during the current study.
