# Supplementary material for: Engineering High-Performance Carbazole-Based Co-Sensitizers: Synthesis, Photophysical Characterization, and Synergistic Enhancement in Dye-Sensitized Solar Cells
Source: J Fluoresc. 2025 Mar 6;35(10):9113–25. doi: 10.1007/s10895-025-04194-1 (PMC12672796; doi:10.1007/s10895-025-04194-1)
Supplement: Supplementary file 1 — Supplementary Material 1 [file 10895_2025_4194_MOESM1_ESM.docx]

**"Engineering High-Performance Carbazole-Based Co-Sensitizers: Synthesis, Photophysical Characterization, and Synergistic Enhancement in Dye-Sensitized Solar Cells"**

Mariam Eltoukhi^a^, Safa A. Badawy*^a^*, Ahmed A. Fadda^a^, Ehab Abdel-Latif^a^, Mohamed R. Elmorsy^a,b^.

*^a^ Department of Chemistry, Faculty of Science, Mansoura University, El-Gomhoria Street, 35516 Mansoura, Egypt.*

*^b^Department of Chemistry, Faculty of Science, New Mansoura University, New Mansoura, 35712, Egypt.*

*^*^ Corresponding author: E-mail:* [m.r.elmorsy@gmail.com](mailto:m.r.elmorsy@gmail.com).

**Materials**

Alfa Aesar and Sigma Aldrich supplied the starting materials, which included carbazole, 1-bromoheptane, 1-ethyl iodide, sodium borohydride, triphenylphosphine hydrobromide, 18-crown-6, malononitrile and cyanoaceticacid. Prior to usage, all solvents were dried. The melting points were measured in degrees Celsius using a Gallenkamp electric melting point instrument and are uncorrected. Thermo Scientific Nicolet iS10 FTIR spectrometer was used to get IR spectra (KBr), while a Burker NMR spectrometer was used to obtain NMR spectra in DMSO-*d6* at frequencies of 400 and 500 MHz (^1^H-NMR) and 125 MHz (^13^C- NMR). A high-performance twin beam spectrophotometer (T80 series) was used to acquire UV-visible spectra. Thermo Fisher Scientific DSQ II GC/MS with Kratos MS-70 ev was used for mass analyses, while a Perkin Elmer 2400 analyzer was used for elemental analysis. The instruments and DSSC fabrications used are listed in the attached information file.


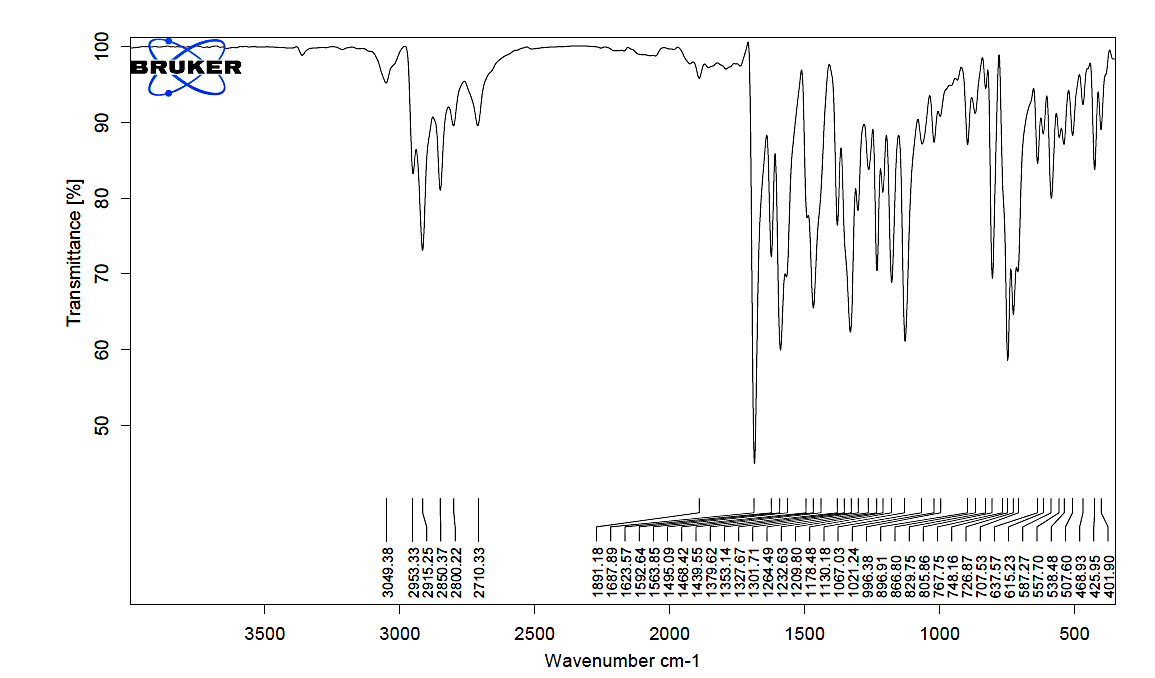

**Fig S1 IR spectrum of compound 7**


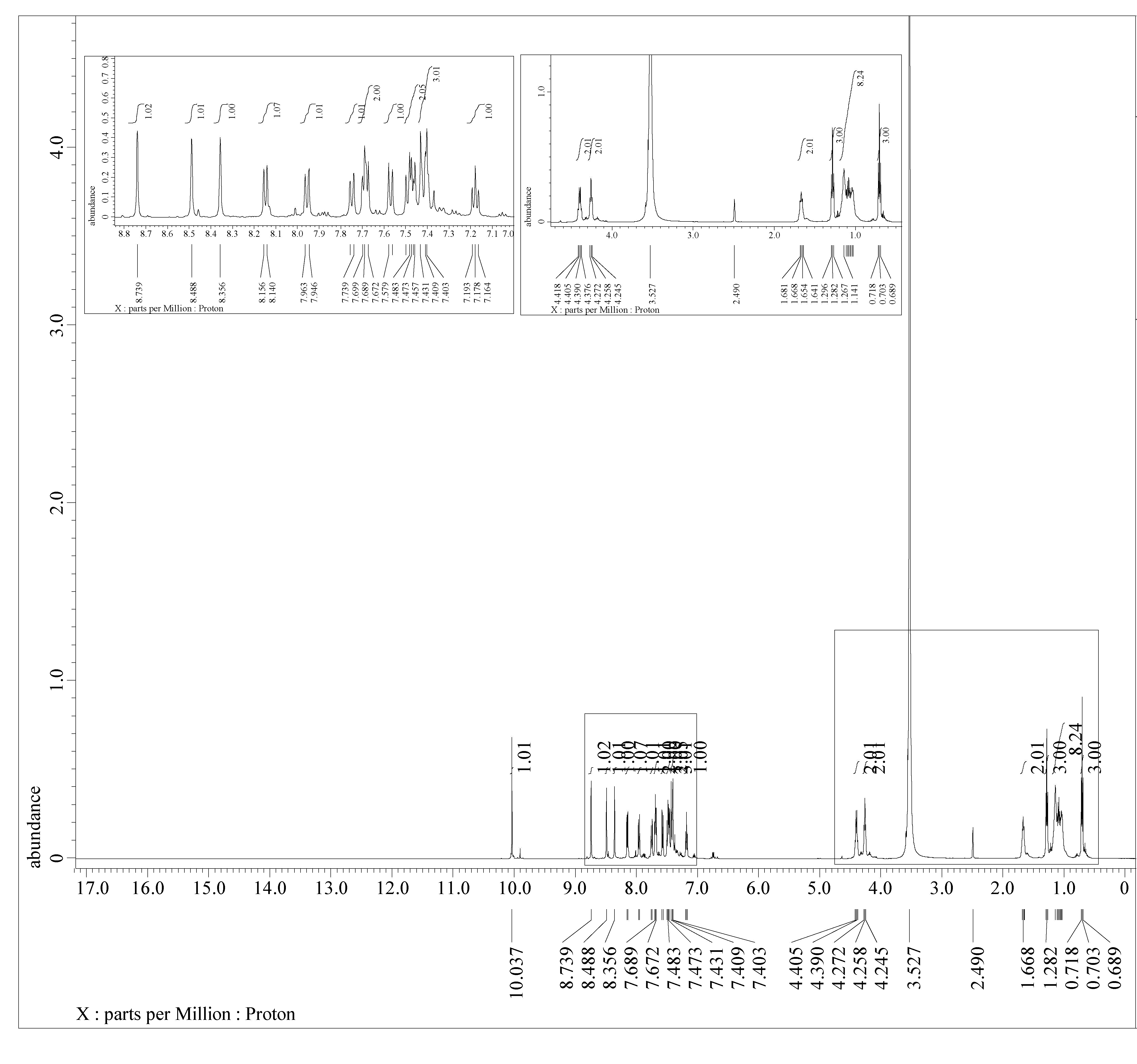

**Fig S2 ^1^H NMR spectrum of compound 7**


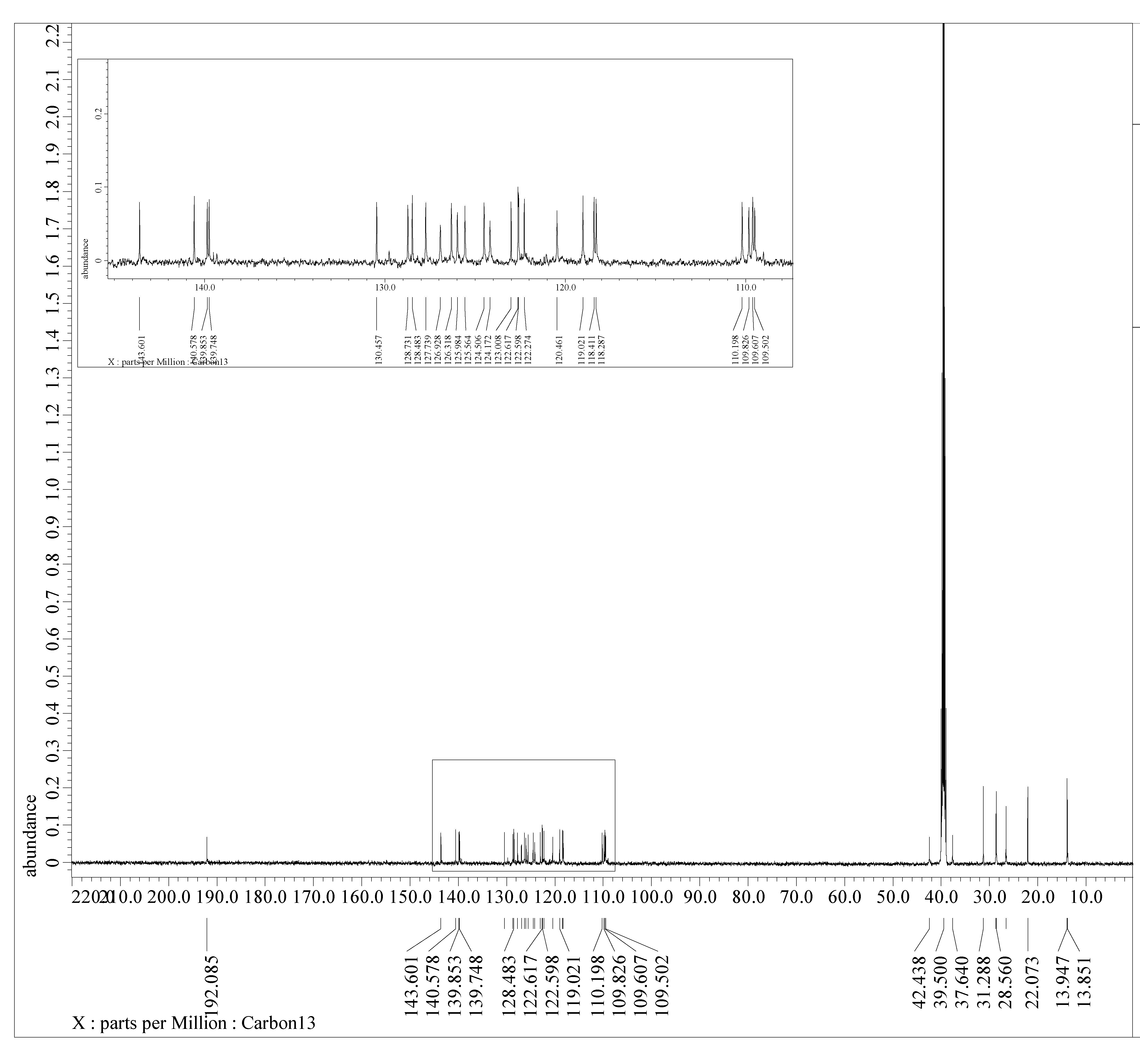

**Fig S3 ^13^C NMR spectrum of compound 7**

**Fig S4 Mass spectrum of compound 7**


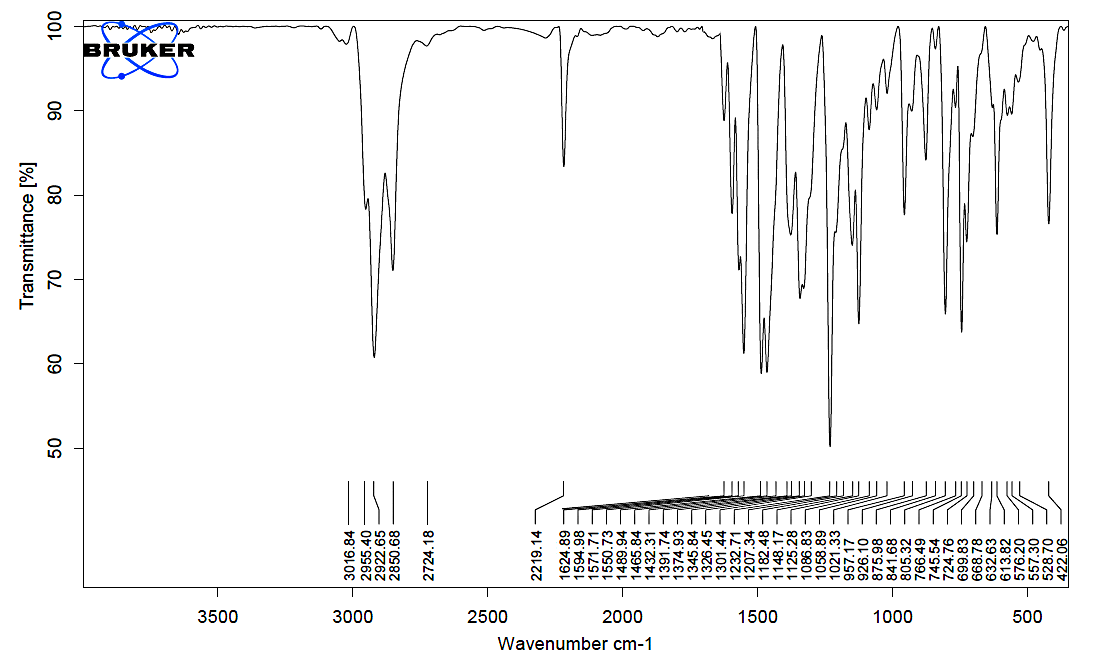

**Fig S5 IR spectrum of compound MA-1**


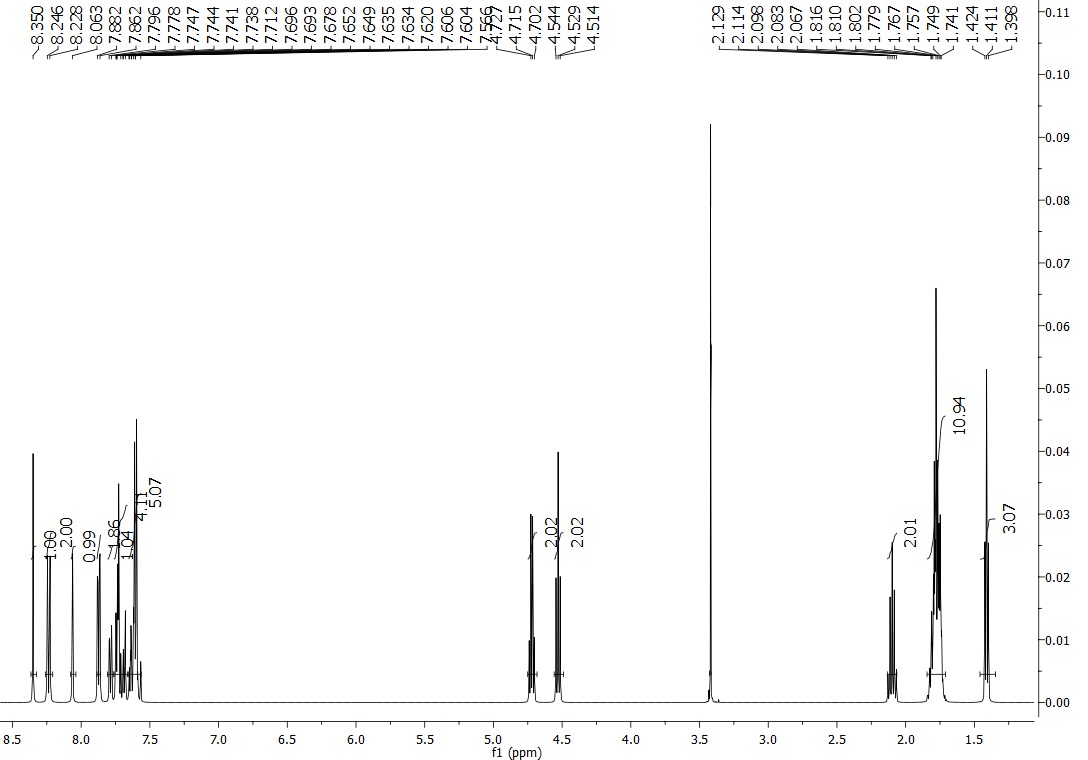

**Fig S6 ^1^H NMR spectrum of compound MA-1**


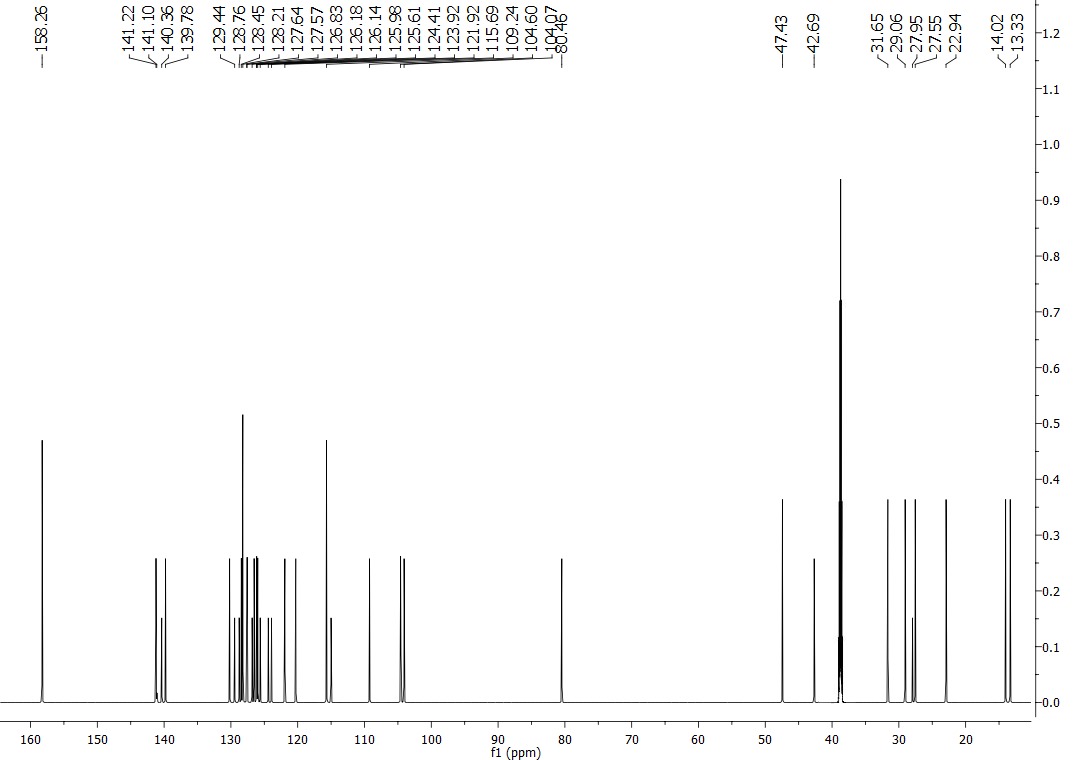

**Fig S7 ^13^C NMR spectrum of compound MA-1**


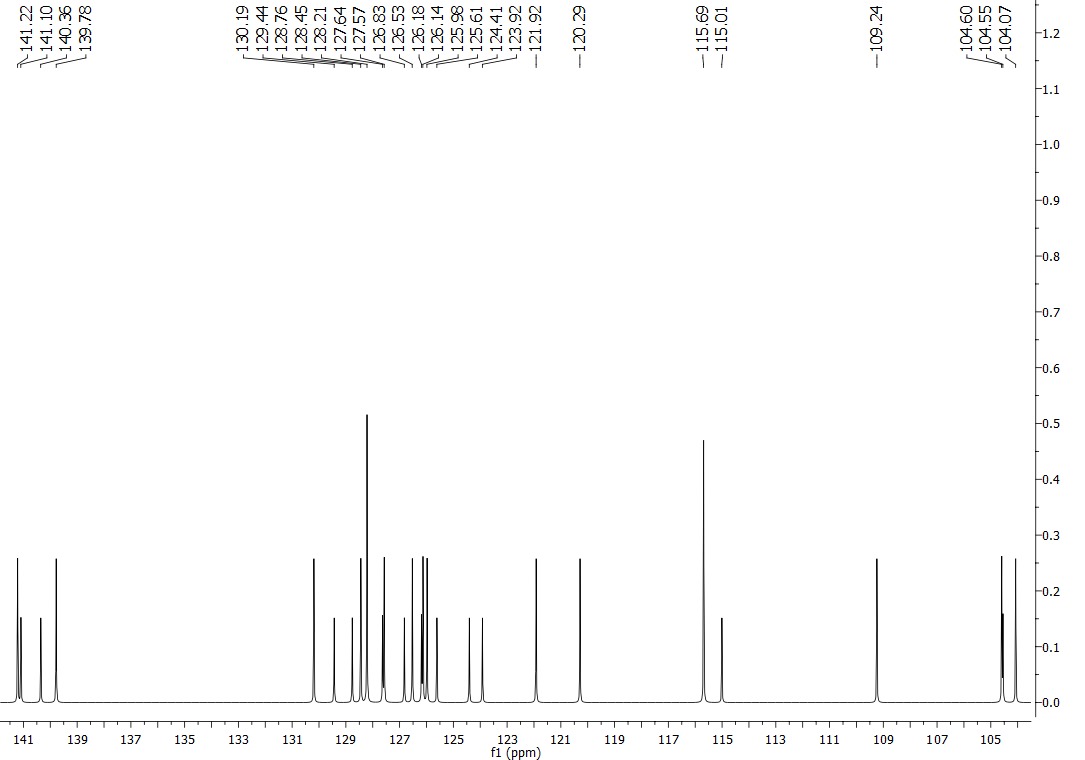

**Fig S8 ^13^C NMR spectrum of compound MA-1**

**Fig S9 Mass spectrum of compound MA-1**


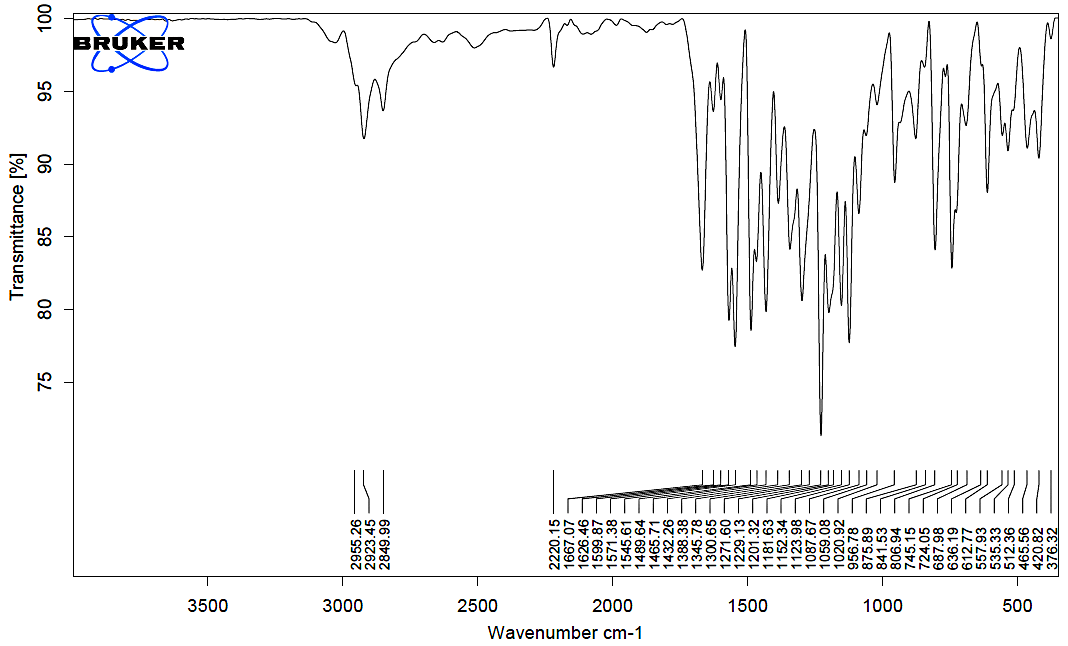

**Fig S10 IR spectrum of compound MA-2**


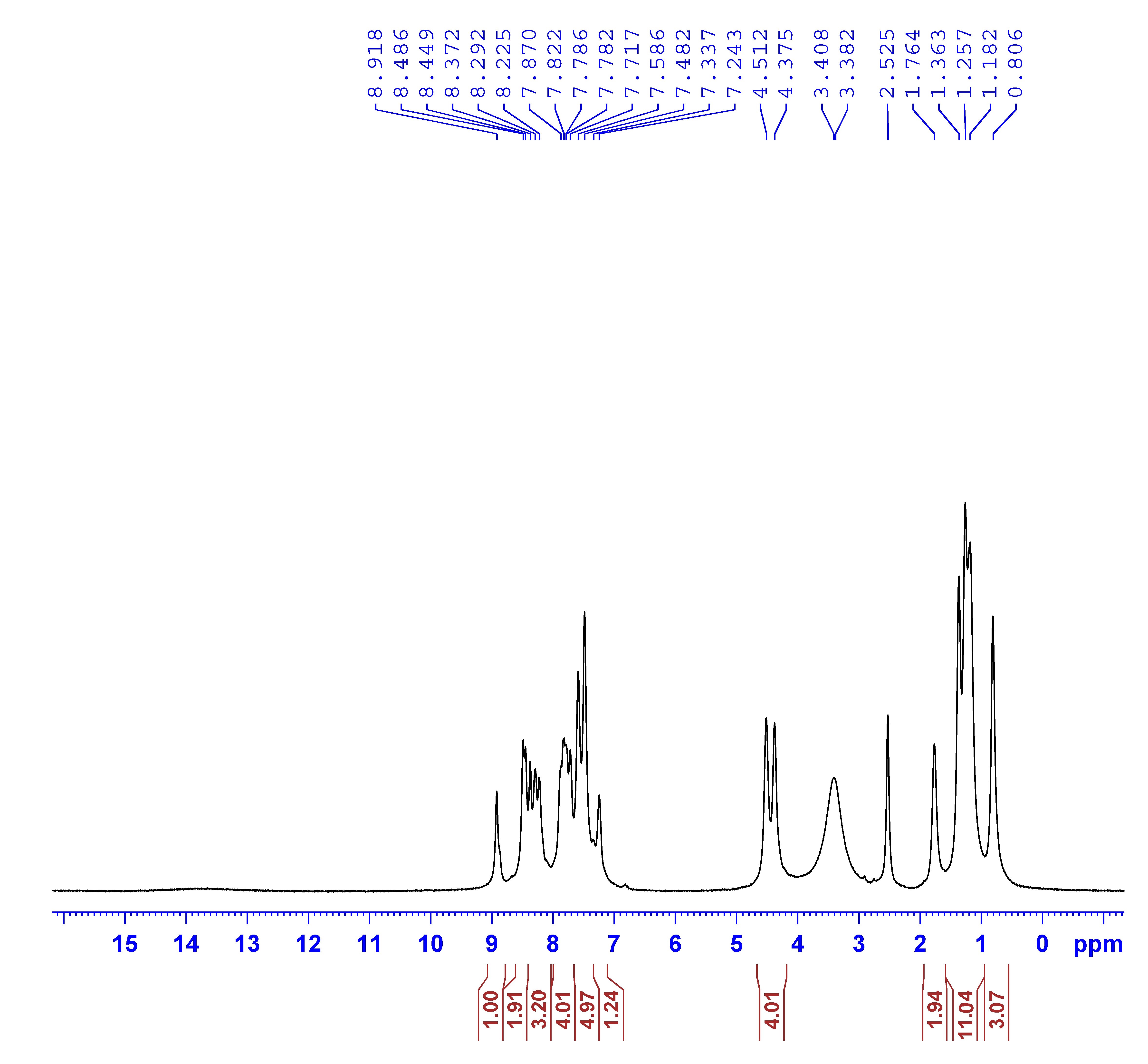

**Fig S11 ^1^H NMR spectrum of compound MA-2**


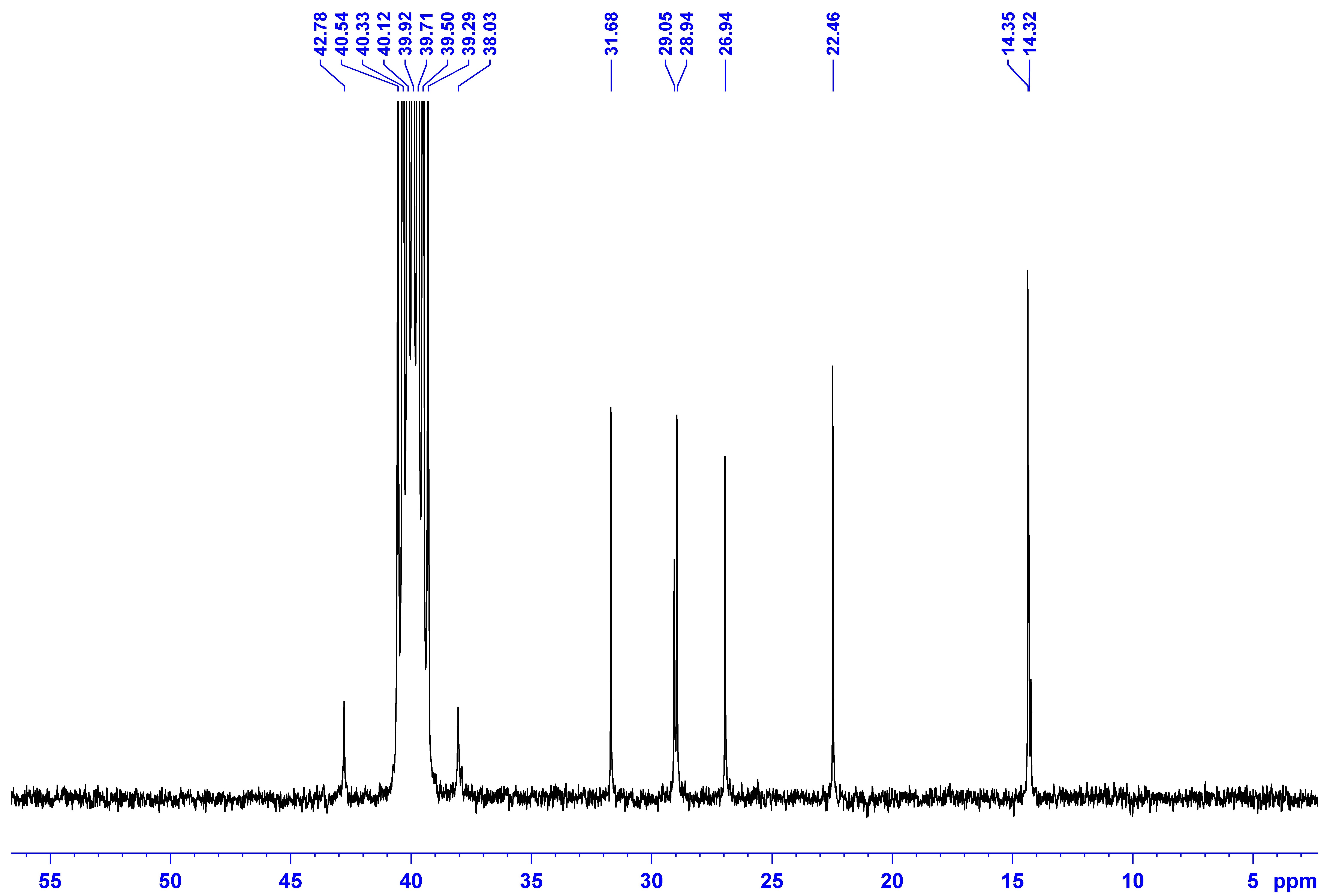

**Fig S12 ^13^C NMR spectrum of compound MA-2**


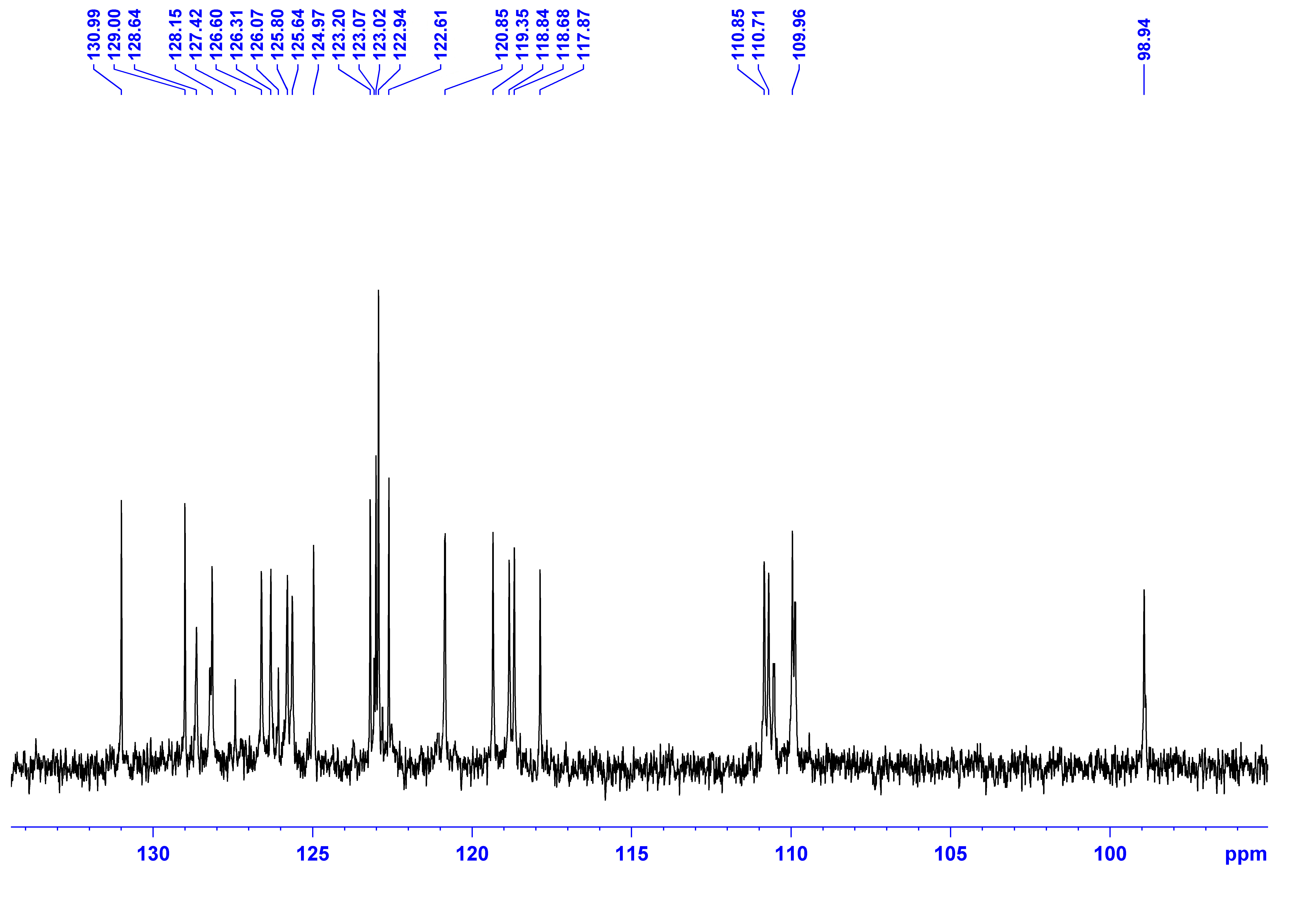

**Fig S13 ^13^C NMR spectrum of compound MA-2**


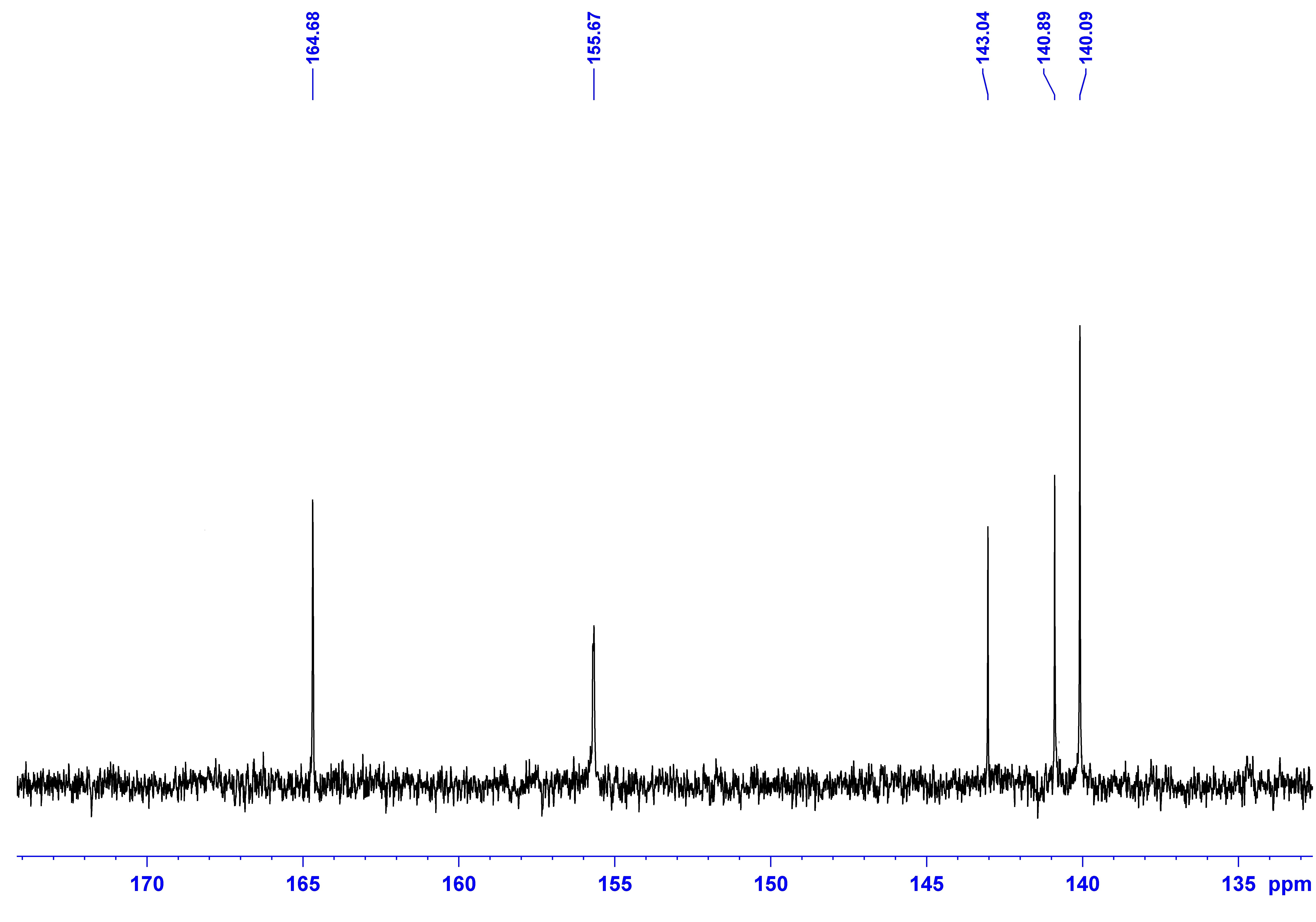

**(Fig S14) ^13^C NMR spectrum of compound MA-2**

**(Fig S15) Mass spectrum of compound MA-2**

**Fig. (S16): Cyclic voltammetry spectra of sensitizers MA-1-2**

**2. Cell preparations and photovoltaic characterizations**

1. **Preparing Electrodes for DSSC**
2. **Working Electrode (Photoanode-TiO_2_ electrode)**

The fabrication process of dye-sensitized solar cells began with the preparation of a TiO_2_ electrode, which consisted of a double-layer structure with a thickness of 10 + 5 μm. The electrode had a 10 mm thick nano-porous layer and a 5 μm thick scattering layer, which were prepared using a previously reported method [1]. The working electrode is subjected to 15 minutes of sonication in a detergent solution, followed by washing with deionized (DI) water, acetone, and ethanol. The electrode is then treated with TiCl_4_ (60 mM) at 90°C for 60 minutes, and subsequently washed with water and ethanol. A layer of nanoporous TiO_2_ (12-14 μm thick) is printed on the electrode using a single 3M transparent tape. The thickness of the layer is adjusted by punching a hole with a 3/16 5.0 stick. A dispersing layer is printed on top of the TiO_2_ layer using a single 3M transparent tape. The thickness of the layer is adjusted by punching a hole with a 1/4 6.0 stick. The TiO_2_ used for the dispersing layer is R/SP. The printed layers are sintered at 350°C for 10 minutes and then at 500°C for 30 minutes. The electrode is then treated with TiCl_4_ at 90°C for 60 minutes and washed with water and ethanol. The electrode is sintered at 500°C for 30 minutes. In the absence of light, the electrode is submerged in a dye solution for a duration of 20 hours. The organic dye solution is comprised of 0.2 mM of each (**MA-1 and MA-2**) in a mixture of acetonitrile, tert-butanol, and DMSO (1:1:1) with addition 0.2 Mm **N3**. Meanwhile, the ruthenium dye solution **(N3**) consists of 0.2 mM of the dye (**MA-1 and MA-2**) dissolved in 9 mL solution of consisting of (3 ml) acetonitrile, (3ml) tert-butanol. In the case of co-sensitization, the dye solution consists of a mixture of 0.2 mM of the co-sensitizers (**MA-1 and MA-2**) and 0.2 mM of the ruthenium dye (**N3**) in a 9 mL solution consisting of (3 ml) acetonitrile, (3ml) tert-butanol. The performance of the dye-sensitized solar cells was characterized by photovoltaic measurements of sealed cells were made by illuminating the cell through the conducting glass from the anode side with a solar simulator at AM 1.5 illuminations (light intensity: 100 Mw.cm^−2^)
**The preparation of a counter electrode involves the following steps:**

The electrode is washed with water, followed by a wash with a 0.1M HCl solution in ethanol (0.2 mL of concentrated HCl in 100 mL of ethanol). The electrode is then subjected to 10 minutes of sonication in an acetone bath. The electrode is dried at 400°C for 15 minutes. A layer of Pt-paste is printed on the electrode using a single 3M transparent tape. The thickness of the layer is adjusted by punching a hole with a 3/8 10.0 stick. The Pt-paste used is Platisol T/SP. The printed layer is cured at 450°C for 10 minutes.

[1] Gad, E. A., Kamar, E. M., & Mousa, M. A. (2020). Experimental and computational study on electronic and photovoltaic properties of chromen-2-one-based organic dyes used for dye-sensitized solar cells. *Egyptian Journal of Petroleum*, *29*(2), 203-209

**2.2. Fabrication of dye-sensitized solar cell**

Photovoltaic and incident photon-to-current efficiency (IPCE) measurements were made on sandwich cells, which were prepared using TiO_2_ coated working electrodes and platinum coated counter electrodes and were sealed using a 40 μm Syrlyn spacer through heating of the polymer frame. The redox electrolyte (Solaronix, Iodolyte HI-30) consisted of a solution of 0.6 M DMPII, 0.05 M I_2_, 0.1 M LiI and 0.5 M TBP in acetonitrile.

**2.3. Photovoltaic measurements**

Photovoltaic measurements of sealed cells were made by illuminating the cell through the conducting glass from the anode side with a solar simulator (WXS-155S-10) at AM 1.5 illuminations (light intensity: 100 mW cm^−2^).

**2.4. Incident photon to current efﬁciency (IPCE) conversion**

IPCE measurements were made on a CEP-2000 system (Bunkoh-Keiki Co. Ltd.). IPCE at each wavelength was calculated using Equation 1, where *I_SC_* is the short-circuit photocurrent density (mA. cm^−2^) under monochromatic irradiation, q is the elementary charge, λ is the wavelength of incident radiation in nm and P0 is the incident radiative flux in W/m^2^.

$$\mathrm{IPCE}\left( \lambda\right)=1240\left( \frac{I_{\mathrm{SC}}}{q\lambda P_{o}} \right) (\mathbf{1})$$

**2.5. Electrochemical impedance spectroscopy (EIS)**

The electrochemical impedance spectra were measured with an impedance analyzer potentiostat (Bio-Logic SP-150) under illumination using a solar simulator (SOL3A, Oriel) equipped with a 450 W xenon lamp (91160, Oriel). EIS spectra were recorded over a frequency range of 100 mHz to 200 kHz at room temperature. The applied bias voltage was set at the *V_OC_* of the DSSCs, with AC amplitude set at 10 mV. The electrical impedance spectra were fitted using Z-Fit software (Bio-Logic).

**2.6. Cyclic voltammetry**

Cyclic voltammetry (CV) was performed in DMF with the electrolyte 0.1 M [TBA][PF_6_] at a scan rate of 50 mV s^−1^. The working electrode used is the Glassy carbon, Pt wire represented the counter electrode and the reference electrode is Ag/Ag^+^ in ACN. Fc/Fc^+^ was introduced as internal reference.

**3. Molecular Modeling**

Equilibrium molecular geometries of **MA-1-2** calculated using the Becke's three parameter hybrid functional, Lee–Yang–Parr's gradient corrected correlation functional (B3LYP) and (6-311g(d, p)) [1, 2, 3, 4]. The geometry optimization calculations were followed by energy calculations using time-dependent density functional theory (TD-DFT) utilizing the energy, functional B3lyp and the basis set 6-311g (d, p). The solvent (DMF) effect was accounted for by using the conductor-like polarizable continuum model (C-PCM), implemented in Gaussian 09.

**References**

[1] G. Melikian, F. Rouessac, C. Alexandre, Synth Commun 23 (1993) 2631.

[2] A. D. Becke, Phys. Rev. A 38 (1988) 3098.

[3] C. T. Lee, W.T. Yang, R.G. Parr, Phys. Rev. B. 37 (1988) 785.

[4] N. Godbout, D.R. Salahub, J. Andzelm, E. Wimmer. Can. J. Chem.-Rev. Can. Chim. 70 (1992) 560-571.
